# G-stack modulated probe intensities on expression arrays - sequence corrections and signal calibration

**DOI:** 10.1186/1471-2105-11-207

**Published:** 2010-04-27

**Authors:** Mario Fasold, Peter F Stadler, Hans Binder

**Affiliations:** 1 Interdisciplinary Centre for Bioinformatics, University Leipzig, Germany; 2 Leipzig Interdisciplinary Research Cluster of Genetic Factors, Clinical Phenotypes and Environment (LIFE), University Leipzig, Germany; 3 Max-Planck-Insitute for Mathematics in Sciences, Leipzig, Germany; 4 Fraunhofer Institut for Cell Therapy and Immunology, Leipzig, Germany; 5 Institute for Theoretical Chemistry, University of Vienna, Vienna, Austria; 6 The Santa Fe Institute, Santa Fe, New Mexico, USA

## Abstract

**Background:**

The brightness of the probe spots on expression microarrays intends to measure the abundance of specific mRNA targets. Probes with runs of at least three guanines (G) in their sequence show abnormal high intensities which reflect rather probe effects than target concentrations. This G-bias requires correction prior to downstream expression analysis.

**Results:**

Longer runs of three or more consecutive G along the probe sequence and in particular triple degenerated G at its solution end ((*GGG*)_1_-effect) are associated with exceptionally large probe intensities on GeneChip expression arrays. This intensity bias is related to non-specific hybridization and affects both perfect match and mismatch probes. The (*GGG*)_1_-effect tends to increase gradually for microarrays of later GeneChip generations. It was found for DNA/RNA as well as for DNA/DNA probe/target-hybridization chemistries. Amplification of sample RNA using T7-primers is associated with strong positive amplitudes of the G-bias whereas alternative amplification protocols using random primers give rise to much smaller and partly even negative amplitudes.

We applied positional dependent sensitivity models to analyze the specifics of probe intensities in the context of all possible short sequence motifs of one to four adjacent nucleotides along the 25meric probe sequence. Most of the longer motifs are adequately described using a nearest-neighbor (NN) model. In contrast, runs of degenerated guanines require explicit consideration of next nearest neighbors (GGG terms). Preprocessing methods such as vsn, RMA, dChip, MAS5 and gcRMA only insufficiently remove the G-bias from data.

**Conclusions:**

Positional and motif dependent sensitivity models accounts for sequence effects of oligonucleotide probe intensities. We propose a positional dependent NN+GGG hybrid model to correct the intensity bias associated with probes containing poly-G motifs. It is implemented as a single-chip based calibration algorithm for GeneChips which can be applied in a pre-correction step prior to standard preprocessing.

## Background

**Figure 1:**
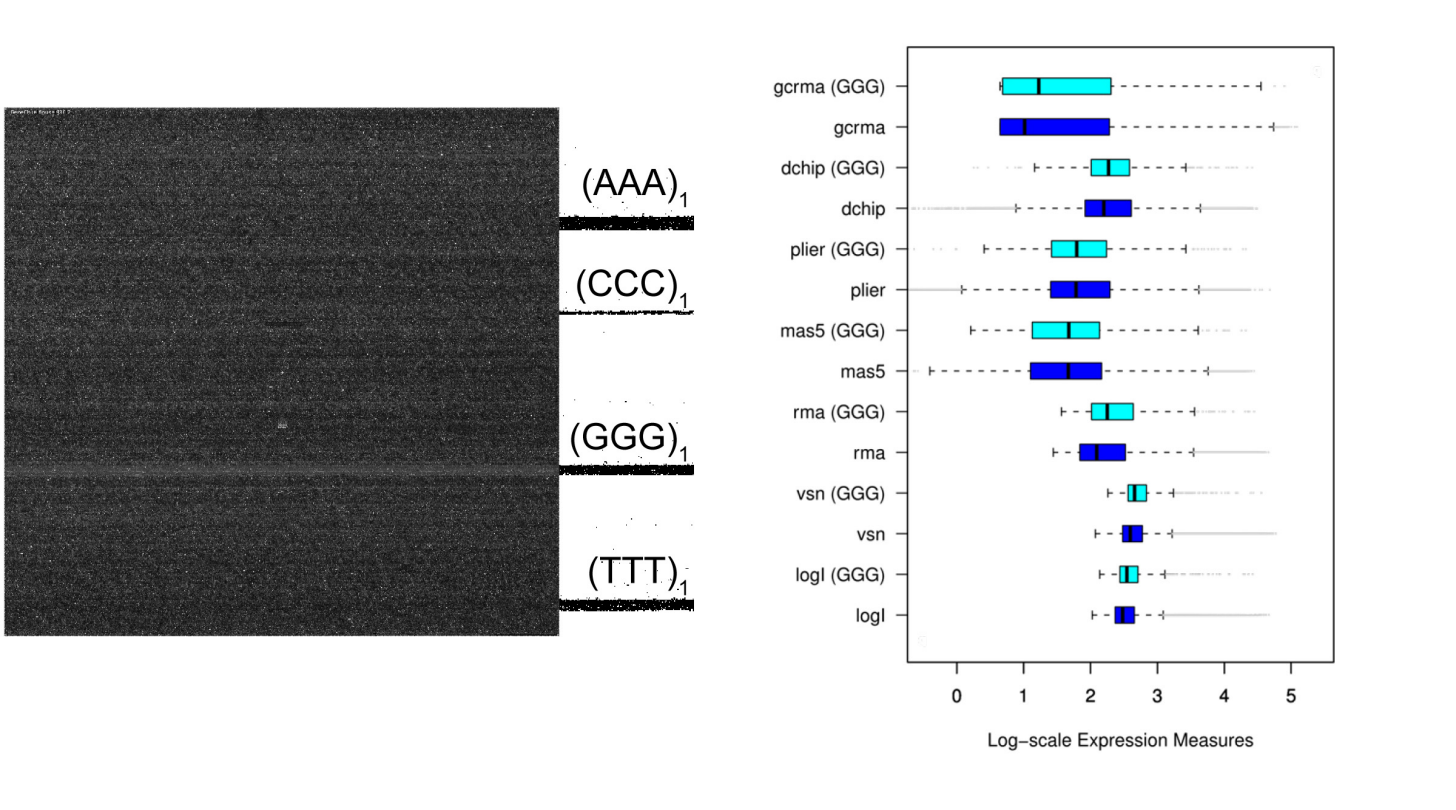
**(a) Fluorescence image of a hybridized Affymetrix GeneChip Mouse Genome MG430 2.0 array (GEO GSE12545)**. The chip surface divides into a grid of nearly 10^6 ^fluorescing probe spots. The bright horizontal stripe matches with probes the 25meric sequence of which starts with triple degenerated guanines ((*GGG*)_1_-motif). Triple runs of other nucleotides are not associated with bright stipes. The position of the respective probes are shown at the rigth border of the figure. (b) Boxplots of expression measures obtained from the intensity data shown in panel a using various preprocessing methods (see text for assignments). The boxplots are computed separately for all probe sets (45,100) and for probe sets with at least two probes containing the (*GGG*)_1_-motif (836, i.e. 2% of the total number). 'log I' denotes the distributions of raw intensity data. Note that essentially all methods except Plier and partly mas5 are unable to correct expression values for the (*GGG*)_1_-bias. The respective distributions of probe sets containing (*GGG*)_1_-probes are systematically shifted towards larger expression values compared with the distribution of all probe sets.

Fig. 1a shows the surface image of a hybridized Affymetrix GeneChip expression array. Its area of about 1.6 cm^2 ^divides into a grid of nearly one million probe spots of size (11 × 11) μm^2^. Each of them is covered by a 'turf' of 25meric oligonucleotides attached to the chip surface. Their sequence is chosen to match complementary oligonucleotide targets which carry fluorescent labels. These targets can be prepared either from messenger RNA (mRNA) to explore the transcriptome in terms of gene and exon expression; or from genomic DNA (gDNA) to discover the genome in terms of protein/DNA interactions, copy number variations and single nucleotide polymorphism (SNP) genotyping. When present, these targets are assumed to bind to the respective probe oligomers giving rise to a bright spot at the chip image. If absent the respective probe spot is assumed to remain dark. Hence, the array experiment translates the abundance of tens of thousands of different targets into an image of intensity spots. In the case of expression arrays the measured intensities thus provide a snapshot of the transcriptional activity of the studied biological sample. The shown image clearly reveals dark and bright horizontal stripes which correlate with the non-random arrangement of probe sequences on the chip: Firstly, the vertical position of perfect match probes (PM) alternates with that of paired mismatch (MM) probes. The intensity of the former ones exceeds that of the latter ones on the average due to their altered middle base which mismatches the target. Secondly and more importantly, the probe sequences arrange in rows with respect to short motifs. In particular, the position of most of the probes possessing triple degenerated guanines at the solution end of their sequence ((*GGG*)_1_) are found within a horizontal band which exactly matches the brightest stripe of the chip image. The respective intensities exceed the average intensity level of the array typically by a factor of two to ten. It seems unlikely that these strong intensity values are associated with extraordinary large expression levels of the respective target genes. Instead the bright intensities can be attributed to probe effects which typically reflect the sequence specifics of probe/target interactions [1]. Such probe effects must be removed from the data to obtain adequate expression values. This obligate correction of raw intensities prior to downstream expression analysis is called calibration or preprocessing. Numerous preprocessing algorithms are presently available to transform raw intensity data into expression measures (for example, vsn [2], RMA [3] and gcRMA [4], dChip [5], MAS5 [6], Plier [7]; see, e.g., ref [8] for a mini-review).

On GeneChip arrays a certain number of probes (usually 11) are collected into one probe set which interrogates the same target gene. Preprocessing summarizes the probe intensities of each probe set into one expression value. Fig. 1b shows the distribution of expression values obtained after calibration of the intensity data shown in panel a using different preprocessing methods (details are discussed below). The boxplots are calculated alternately either for all probe sets of the array or for probe sets which contain at minimum two (*GGG*)_1 _probes. The results obtained from most of the preprocessing methods clearly reveal a systematic shift of the expression values of this (*GGG*)_1 _sub-ensemble to higher levels. These calibrations obviously fail to correct the strong intensity bias properly.

As one option to solve this problem one can simply exclude the 'bad' (*GGG*)_1 _probes from further analysis. However, we show below that also other motifs, for example runs of degenerate guanines along the whole sequence, can cause systematic intensity biases. The masking of such 'bad' probes will exclude a significant fraction of the available intensity data from expression analysis and thus reduce the information potentially available from the microarray experiment. We suggest therefore an alternative strategy which intends to correct the probe-related intensity effects. It aims at extracting the 'hidden' information about target abundance in terms of corrected intensities for further use in downstream analysis.

This contribution addresses this issue and presents a systematic study of the effect of short sequence motifs of up to four adjacent nucleotides at all possible sequence positions on the resulting probe intensity. We compare the results obtained for different hybridizations after variation of the sample RNA, chip type and/or the amplification protocol. Our approach aims at identifying the minimum motif length for appropriate intensity prediction using a positional and motif dependent model. We focus on the effect of runs of degenerated guanines which have been found to behave unusually compared with other motifs in different chip assays including Affymetrix expression and SNP arrays [9-14].

The remainder of the paper is laid out as follows. In Section 2, public available microarray intensity data are analyzed in terms of positional dependent models of rank 1 to 4 using single base (N), nearest neighbor (NN), next nearest neighbors (NNN) and quadruple (NNNN) sequence motifs. We assay experimental factors such as the chip type, the sample RNA and the amplification protocol, disentangle the role of specific and non-specific hybridization and compare PM and MM probes. Based on the results of these analyses we propose a NN+GGG hybrid model to correct the probe intensities for sequence effects. An implementation of the method can be downloaded from our website http://www.izbi.de. The discussion in Section 3 compares the performance of different preprocessing algorithms with respect to sequence effects and the potency of different sequence models for use in intensity calibration for microarrays. The methodical Section at the end of the paper sets out the basic equations and background theory for the analysis and provides also references for the used array data. The supporting material supplements the main results given in the paper [Additional file 1].

## Results

### Positional dependent sensitivity profiles of different rank

**Figure 2:**
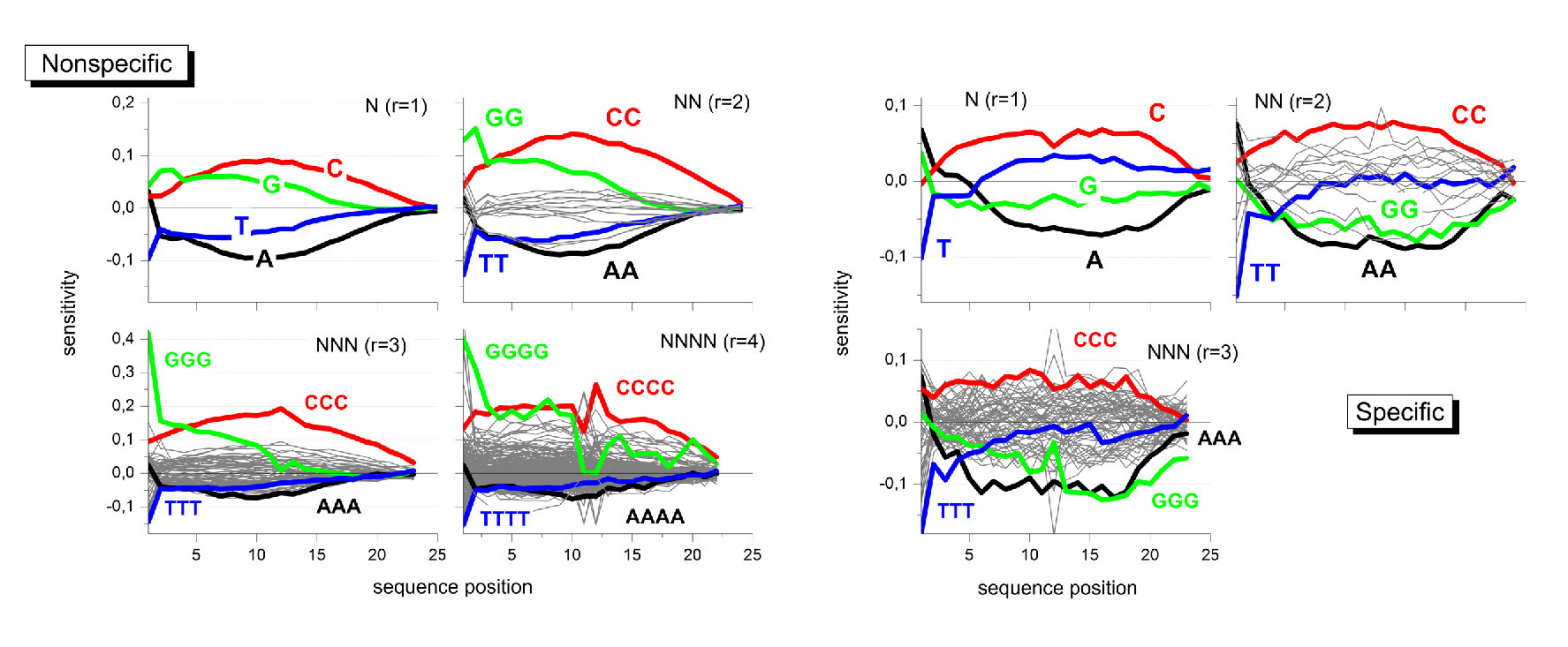
**Positional dependent sensitivity profiles of different rank for non-specific (left) and specific (right) hybridization computed from the Mouse-dataset shown in Fig. 1**. Runs of equal bases (e.g. AAA) are emphasized by thick lines in different colors. Note the different ranges of the ordinate scales in both rows of the figure.

We apply the positional dependent sensitivity model descibed in the methodical section below to the intensity data shown in Fig. 1a. The model provides sensitivity profiles of rank 1 to 4, the maximum rank being limited by the available number of data points. Fig. 2 (left part) shows the profiles which were obtained using the intensities of 'absent' called PM probes. The sensitivity terms can be interpreted as the logged intensity increment due to the respective sequence motif of *r *consecutive bases starting a position *k *of the 25meric sequence (see subsections 5.2 - 5.6).

The shapes of the four single base profiles (*r *= 1) virtually agree with previously published data [15-19]: The sensitivities of adenines (A) and cytosines (C) are roughly symmetrical with respect to the x-axis and change in a parabola-like fashion, the maximum being near the middle of the probe sequence. The profiles of guanine (G) and thymine (T) indicate a more monotoneous dependence. All profiles are asymmetrical with respect to the ends of the probe sequence: They converge towards the surface-attached side at *k *= 25 but differ significantly near the solution end at *k *= 1. The sensitivities and thus the base- and positional dependent contribution to the intensities increase according to *A *<*T *<*G *<*C *for most sequence positions. The nearest neighbor model (*r *= 2) provides a total of 16 profiles. Most of them relatively tightly group about the x-axis resembling essentially the parabola-like shape of the single base profiles for A and C. The contributions of the CC-profile however markedly inflates for all sequence positions whereas the GG-profile increases especially at small k. This latter trend is partly counterbalanced by negative values of T-containing NN-termns, especially of TT.

Inspection of the 384 NNN-profiles (*r *= 3) shows that these trends further intensify for stacks of cytosines (CCC) and guanines (GGG) where the relative level of the latter profile increases compared with that of CCC.

G-quadruples clearly dominate at small position indices *k *< 4 among the 1512 quadruple profiles of rank *r *= 4. Also other motifs indicate a relatively strong contribution at small *k *as well (e.g. GCCC and GGGA). The contribution of GGGG-quadruples (and of other triple-G containing motifs) markedly drops for *k *> 13, i.e. for positions closer to the surface end of the probes. Note also, that the parabola-like shape of the profile of runs of adjacent cytosines changes into a broad plateau which decreases only near the ends of the probe sequence.

Hence, the contribution of a few motifs, especially of degenerated runs of C and G but also of selected GC-rich tuples, increases above average with the extension of the model rank from *r *= 1 to *r *= 4: more than twofold for CCCC and up to tenfold for GGGG compared with the respective single nucleotide values. Longer homo-motifs obviously adapt to specific intensity effects.

The sequence effect of some of the motifs reaches its maximum in the middle of the sequence. With increasing model rank, these peaks reshape into a broad plateaus of virtually constant sensitivity values which markedly change only near the ends of the probe sequence. In contrast, G-rich subsequences add strong intensity contributions at small position indices especially at the first sequence position. The respective contributions progressively increase for *r *= 1 to 3 but then remain virtually unchanged for *r *= 4. Note that also the guanine profiles of lower rank (G, GG and GGG), show exceptional large positive values at sequence positions *k *< 4. The possible origin of this behavior will be discussed below.

The right part of Fig. 2 shows the corresponding profiles of the PM probes predominantly with specific hybridization. Only 8% - 20% of all probes on the chip meet this criterion. This relative small number of probes restricts the rank of the model to *r *= 1 - 3 and, moreover, gives rise to a relatively large level of noise. The specific profiles possess essentially the same properties as the non-specific ones shown in the left part of Fig. 2 except that for G- and T-rich motifs. In particular, profiles of homo-runs of guanines shift markedly towards smaller values compared with their non-specific values. Note also that the (*GG*)_1 _and especially (*GGG*)_1 _motifs at the solution end contribute much less to the specific profiles.

### Guanine effects

**Figure 3:**
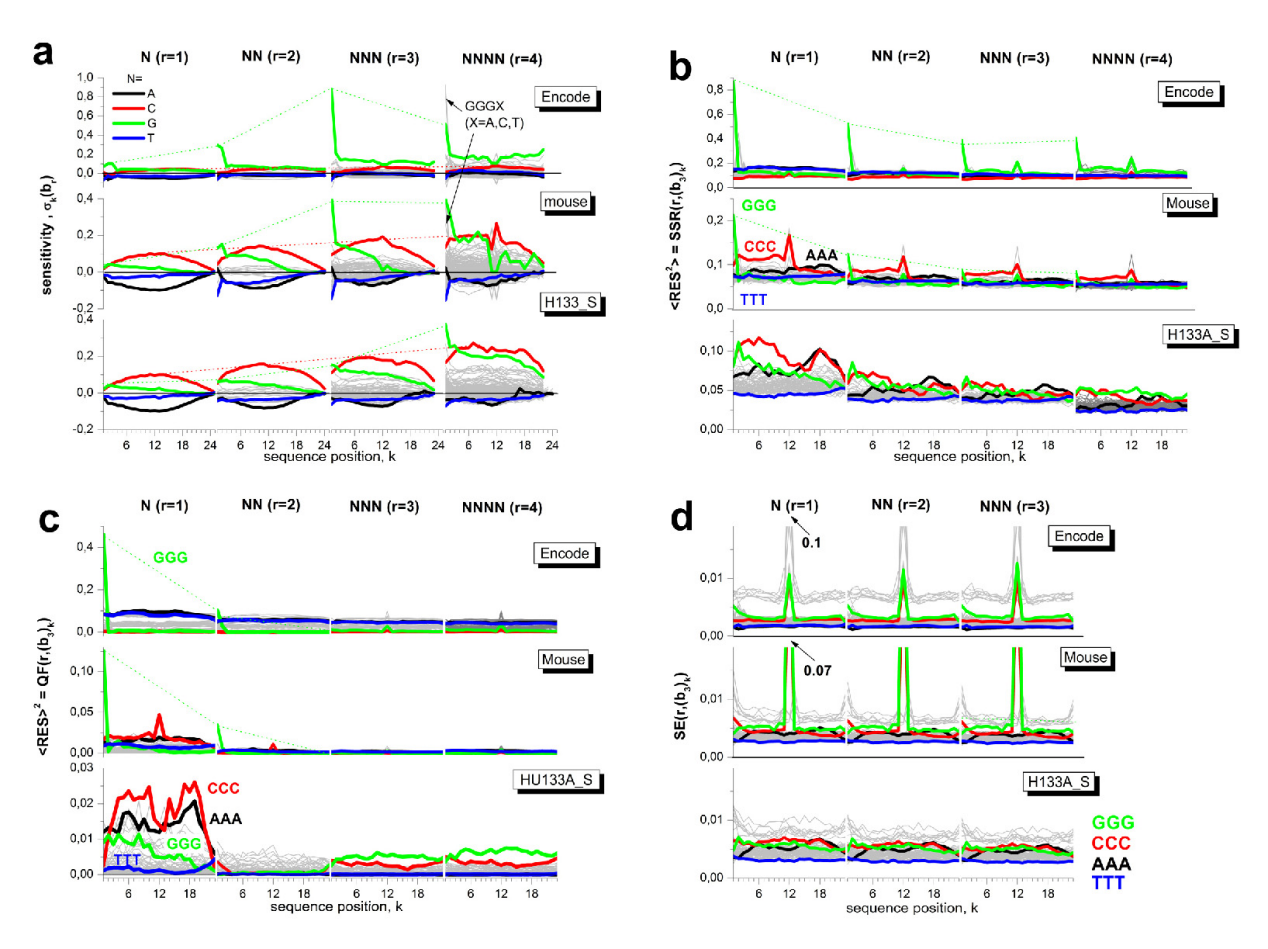
**Sensitivity profiles of rank *r *= 1 - 4 of different data sets (part a, see Table 2 in the methodical part) and the respective triple-related fit statistics: sum of squared residuals (part b, Eq. (8)), quality of fit (c, Eq. (13)) and standard error (d, Eq. (14))**. Homo-motifs of consecutive A, C, G and T are shown by colored curves. The thin dotted lines indicate the basic trends of the poly-G and poly-C motifs at position *k *= 1 and 12, respectively. Note the different scaling of the ordinates in panels a and c.

**Figure 4:**
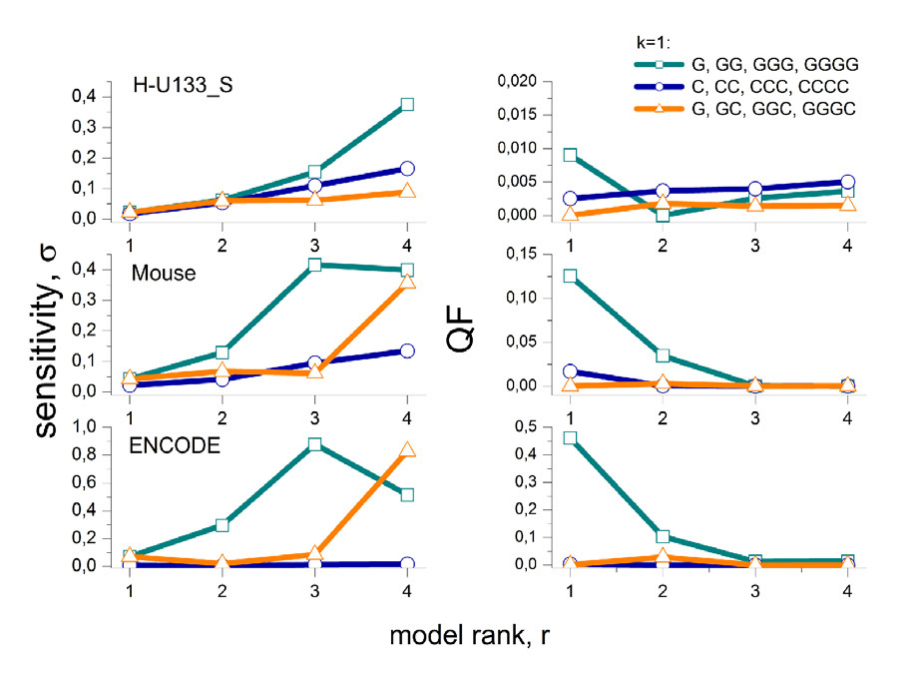
**Sensitivity terms and quality of fit of selected motifs of rank r at position k = 1 of the probe sequence**. The data are replotted from Fig. 3 part a and c. The fact that the sensitivity of degenerated G levels off for *r *> 2 whereas that of GGGC steeply increases for the ENCODE and mouse data set indicated the strong (*GGG*)_1_-effect. The respective quality of fit reaches acceptable values only for *r *> 2 which indicates that at least runs of three guanines must be explicitly considered.

Part a of Fig. 3 compares the sensitivity profiles of non-specifically hybridized probes of the mouse data set shown in Fig. 2 with the respective profiles of the ENCODE and HG133A_S data sets. As a general trend, the sensitivity level of poly-C terms nearly linear increases with increasing rank of the model as indicated by the dotted lines. This trend reflects a constant incremental contribution per additional cytosine in the considered motifs. In contrast, the sensitivity of poly-G motifs starting at *k *= 1 steeply gains at *r *= 3 (ENCODE and mouse data sets) or, to a less extend, at *r *= 4 (HG133A_S data set). We re-plot the respective sensitivity values in the left part of Fig. 4. They reflect an extraordinary strong intensity increment due to three consecutive guanines starting at the first sequence position in the former situation and due to four consecutive guanines along the whole probe sequence in the latter situation. We will call these properties shortly (*GGG*)_1_- and poly-G effect, respectively.

The (*GGG*)_1_-effect is further supported by similar values of the sensitivity terms for quadruples starting at *k *= 1 with threefold degenerated guanines *GGGB *(*B *= *A, T, G, C*; see the arrows in Fig. 3) and that of the respective triple-G, i.e. *σ*_1_(*GGG*) ≈ *σ*_1_(*GGGB*) (see Fig. 4). It shows that the (*GGG*)_1_-motif adds the dominating intensity contribution to that of the *GGGB*-quadruples.

Note that the (*GGG*)_1_-effect of the ENCODE-data set largely exceeds that of the mouse data set by nearly one half order of magnitude: An initial run of three G increases the intensity relative to the mean intensity level by the factor of 10^1.0 ^= 10 and 10^0.4 ^= 2.5 in the former and latter data set, respectively. The intensity increment due to a triple-C motif in the middle of the probe sequence is distinctly smaller and amounts to a factor of about 10^0.2 ^= 1.6. Subtle differences between the sensitivities due to the different hybridization chemistries (DNA/RNA versus DNA/DNA in the mouse and HG133A_S sets versus ENCODE) will be discussed separately below.

In summary, triple degenerated guanines at the solution end of the probe sequences cause exceptionally large intensities in selected data sets. Longer runs of consecutive G along the probe sequence are also associated with large intensities, however to a smaller extend.

### Quality of motif specific fit

The discussed sensitivity profiles were obtained by multiple linear regression fits of Eq. (7) to the intensity data of non-specifically hybridized probes of the respective arrays by minimizing the total sum of squared residuals (SSR) (Eq. (8)). The fit of models of increasing rank *r *improves the goodness of fit in terms of the total *SSR*(*r*) (Eq. (8)). Table 1 lists the total *SSR*(1) values of the single base model and the respective F-values for models of rank *r *= 2 - 4 (Eq. (10)). Maximum improvement is observed for the NN model compared to N and smallest for NNNN compared to NNN.

**Table 1:** Sum of squared residuals of the fits of model ranks *r *= 1 ... 4: *SSR *of all probes and of probes containing C-triples and the (*GGG*)_1_-motif are given for the N-model (*r *= 1).

	HG133A_S	Mouse	ENCODE
*SSR*(1)	0.048	0.072	0.11
*SSR*(1, *CCC*)	0.072	0.088	0.094
*SSR*(1, (*GGG*)_1_)	0.071	0.21	0.85

F: N → NN	147.52	202.08	381.73
F: NN → NNN	11.11	20.6	78.07
F: NNN → NNNN	3.09	6.16	8.89

The respective F-values for the higher ranks *r *= 2 - 3 evaluate the improvement of the fits with respect to the model of next smaller rank *r *- 1.

The total SSR was decomposed into motif and positional dependent terms according to Eq. (12) to characterize the model fits of rank *r *= 1 - 4 more in detail (Fig. 3b). In general, the mean level of the SSR-terms decreases with increasing rank of the model indicating the improvement of the fits in parallel with the decrease of the total SSR discussed above. The partial SSR values of selected motifs (e.g. degenerated cytosines and guanines) are larger than the average level for the N-model. Especially the value of the (*GGG*)_1_-motif largely exceed the total SSR value by nearly one order of magnitude (ENCODE) and by the factor of 2-3 (mouse data set) indicating inadequate fitting of this motif (see Table 1 and Fig. 4). The SSR-values estimate the deviation between the fitted and the experimental data. They can be attributed to two potential origins, namely the systematic bias due to the inadequacy of the model and/or the random scattering of the experimental data. We calculate motif- and positional dependent profiles of the qualitiy of fit (QF, Eq. (13)) and of the standard error (SE, Eq. (14)) as suited measures to estimate the respective contributions. Particularly, one expects vanishing QF-values for adequate fits of the model. The motif and positional data shown in part c of Fig. 3 reveal that the N-model fails fitting the probe intensities of all considered data sets. The NN-model markedly improves the fit for all motifs except (*GGG*)_1_. Clearly this motif gives rise to residual systematic deviance between the fits and the respective intensities of the mouse and ENCODE data sets. It however largely vanishes for *r *= 3. This result confirms our hypothesis that the observed intensity effect is related to threefold degenerated guanines (*GGG*)_1_. The QF-profiles of the HG133A_S data set reveal small systematic deviations of degenerated guanines motifs along the whole sequence for *r *= 3 and 4 due to the poly-G effect.

The standard error is relatively invariant for most of the motifs and positions with *SE *< 0.01 as a rule of thumb (part d of Fig. 3). Note that selected GC-rich motifs in the middle of the probe sequence are very rare on the MG230 2.0 and ENCODE arrays with partly less than 100 probes containing these motifs (the respective number-profiles of triple motifs are given in the supplementary material [Additional file 1]). These small numbers gives rise to imprecise estimates of the respective sensitivity terms.

In summary, the decomposition of the total fit statistics into motif- and positional dependent contributions reveals adequate fits of most of the motifs using the NN-model. As a clear exception, the (*GGG*)_1_-effect requires explicit consideration of NNN-terms for adequate fitting.

### Chip-type and target effects

**Figure 5:**
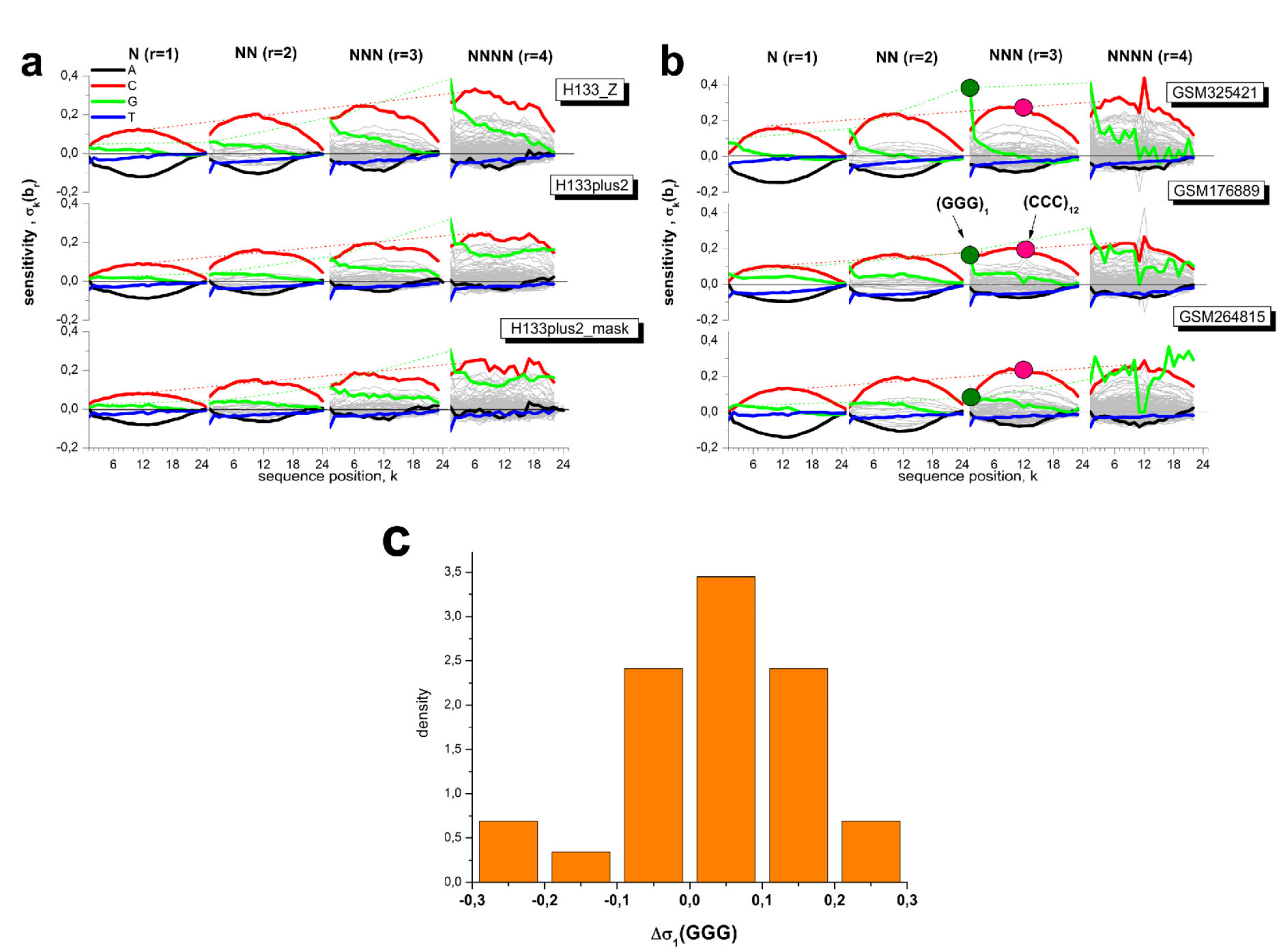
**Chip-type and RNA effect on the sensitivity profiles of rank *r *= 1 - 4**. The profiles refer to different hybridizations which use either identical mRNA (human reference RNA) and different chip types (panel a, HG U133A versus HG U133plus2) or arrays of the same type (MG-230 2.0) but different RNA samples (panel b). The profiles of the 'HG133plus2_mask' analysis (part a) refer to the same collection of probe sequences as used for calculating the HG133_Z profiles. In general, the sensitivity profiles are relatively invariant in the different situations except the amplitude of the (*GGG*)_1_-effect which considerably scatters in different hybridizations to the same chip-type (see part b). Part c of the figure shows the distribution of the relative (*GGG*)_1_-amplitude Δ *σ *(*GGG*) for 29 independent hybridizations to MG-230 2.0 arrays (the list of GEO-accession numbers is given in [Additional file 1]). Δ*σ *(*GGG*) is calculated as the difference between the sensitivity values of (*GGG*)_1_- and (*CCC*)_12_-motifs (see part b for illustration).

The data sets so far address different target samples which are hybridized onto different chip types. Both factors potentially affect the motif and positional dependent sensitivity profiles, and, in particular, the poly-G effects discussed above. To discriminate between effects due to target and chip-type we compare the sensitivity profiles for different hybridizations of the same RNA sample (Universal Human Reference RNA) to two different chip types, namely the newer HG133plus2 and the previous-generation HG 133A array. The nearly 55,000 probe sets of the former chip integrate the more than 22,000 probe sets of the latter one and this way allow direct comparison of the intensity of probes of identical sequences on the two chip types after appropriate masking of the additional probes in the HG133P_Z data set. The obtained three sets of profiles of rank *r *= 1 - 4 are very similar and provide no indication that the two considered chip types strongly modify their shape (see Fig. 5a). For example, the poly-G effect is observed in all three data sets. In the next step we compare the profiles of different RNA-hybridizations to the same chip type (MG430 2.0, see Fig. 5b). Also in this case the profiles of most of the motifs look similar for the different hybridizations except the sensitivity terms of homo-G runs at the first sequence position which indicate different amplitudes of the (*GGG*)_1_-effect. For direct comparison we normalize the respective triple sensitivity term relatively to the maximum sensitivity value of triple-C motifs in the middle of the sequence and calculate the difference Δ*σ *(*GGG*) = *σ*_1_(*GGG*) - *σ*_12_(*CCC*) as a relative measure of the amplitude of the (*GGG*)_1_-effect (see Fig. 5b for illustration). Part c of Fig. 5 shows the distribution of the obtained Δ*σ *(*GGG*)-values for a series 29 independent hybridizations using MG430 2.0 arrays. The data show that the amplitude of the (*GGG*)_1_-effect varies over a wide range for different target hybridizations of the same chip type.

**Figure 6:**
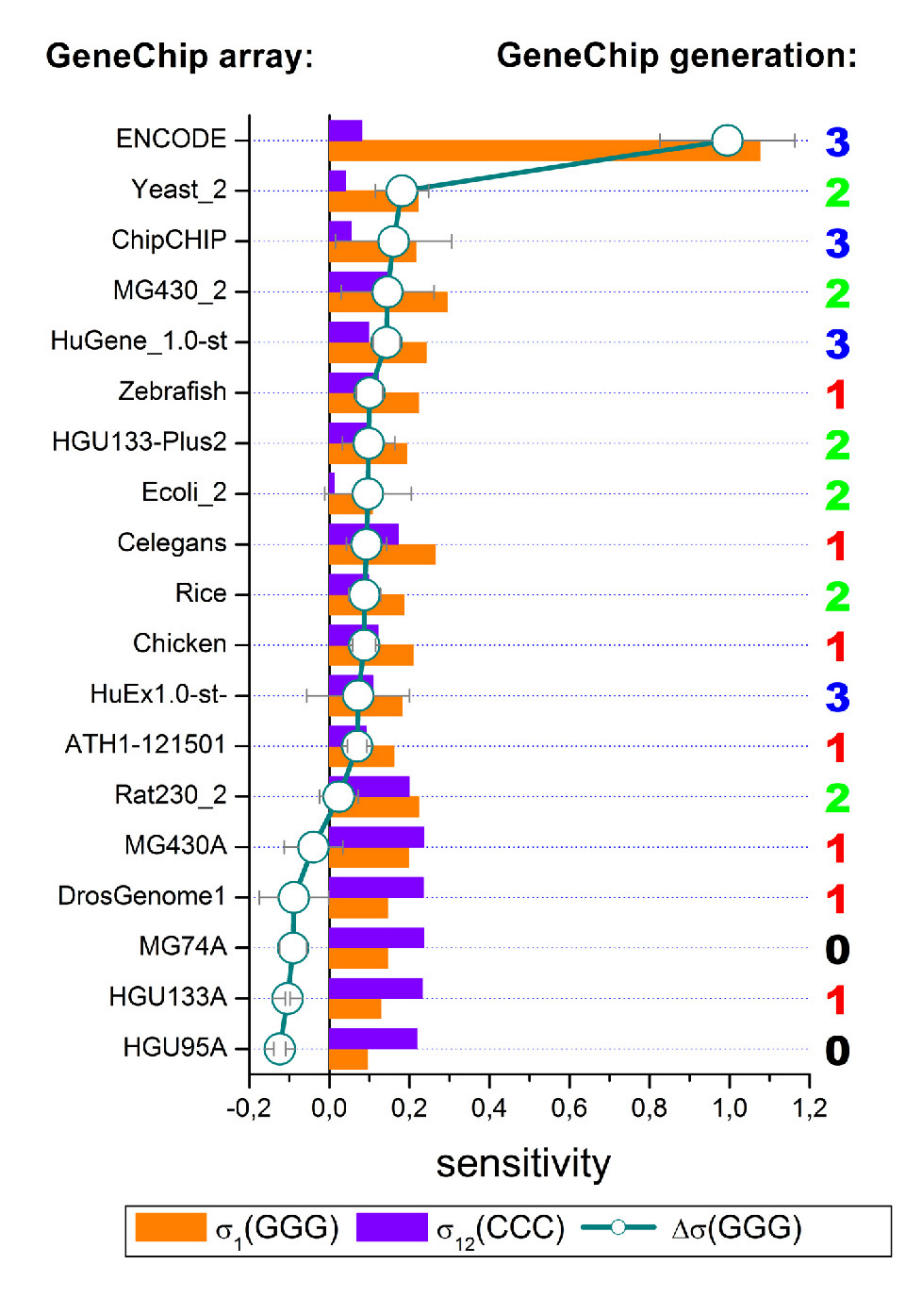
**The amplitude of the (*GGG*)_1_-effect on GeneChips of different type**. The bars refer to the sensitivity terms of triple-G and -C at the first and the middle position of the sequence, respectively. The arrays are ranked with respect to the difference Δ*σ *(*GGG*) which characterizes the amplitude of the (*GGG*)_1_-effect (circles, see text). Sensitivity profiles of three independent hybridizations are averaged for each value. The numbers on the right assign the chip generation 0 to 3 (see text). The amplitude of the (*GGG*)_1_-effect tends to increase with the chip generation. The GEO-accession numbers of the samples analyzed are given in the supplementary material (see [Additional file 1]).

In the next step we estimated the amplitude of the (*GGG*)_1_-effect for eigtheen different array types. Fig. 6 plots the mean sensitivity amplitudes *σ*_1_(*GGG*), *σ*_12_(*CCC*) and their difference. The considered chip types can be roughly classified into four chip-generations (numbered 0 to 3) which use different probe spot sizes, number of probe spots per chip and partly different hybridization chemistries. The spot sizes decrease from 18-20 μm (generations 0 and 1), to 11 μm (generation 2) and to 5 μm (generation 3) which results in the marked increase of the number of probes per chip. Generation 3 (Human Gene 1.0 ST and Human Exon 1.0 ST arrays) uses a PM-only design without MM-probes and DNA/DNA instead of DNA/RNA probe/target-hybridization chemistry. We assign the ENCODE arrays also to generation 3 because it applies DNA/DNA hybridizations as well. However, it still uses MM probes and larger spot sizes (10 μm) compared with Gene 1.0 ST and Exon 1.0 ST arrays. 'ChipChIP' assigns arrays of the ENCODE-type which are applied in ChipChIP experiments. On chips of generations 1 - 3 most of the probes containing (*GGG*)_1_-motifs are located in a row as shown in Fig. 1.

It turned out that the (*GGG*)_1_-effect can be identified for all arrays of generations 1 to 3. Its amplitude tends to increase for chips of later generations 2 and 3. The differences between the chip generations are however moderate without clear indication that type-specific factors such as the arrangement of probes, their spot size, density and number explicitly explain the (*GGG*)_1_-effect.

Interestingly, our data reveal a large difference of the amplitude of the (*GGG*)_1_-effect between the ENCODE-expression and ENCODE-ChipChIP hybridizations (Fig. 6). Both experiments use the same type of ENCODE tiling arrays but different amplification protocols: The former one amplifies sample mRNA via T7-priming and subsequent reverse transcription to double stranded cDNA whereas the latter one amplifies genomic DNA after immunoprecipitation via random priming without the T7-protocol [20-24]. Note that fragments of the T7-primers used in the amplification step of mRNA-sample preparation partly remain bound to the amplified targets as has been discussed in [25]. The respective common G-rich sequence motif of the primer (5'-GGGCGGAGG...) contaminates a large fraction of the targets at their 5'-end and preferentially bind to probes with complementary C-rich motifs [25]. One might hypothize that these fragments are prone to associate also with selected G-rich probe sequences to form mixed probe/target G-quadruplexes which give rise to the strong intensity of the respective probe spots. 

In summary, we found systematic differences between the amplitude of the guanine effects between GeneChips of different generations which are rather gradual than fundamental. On the other hand, our data suggest that the amplification protocol for the used targets strongly affects the (*GGG*)_1_-effect. Previous studies showed that the targets become contaminated with G-rich primer fragments after T7 amplification with possible consequences for their binding affinity to G-rich probes.

### Perfect match and mismatch probes

Each perfect match (PM) probe is paired with one mismatch (MM) probe on most of the Affymetrix microarray types. The MM probes use the same 25meric sequence as the respective PM probes except for the middle base, which is substituted by its complement. To extract subtle differences between the sensitivity profiles of both probe types we calculate the logged intensity difference of each probe pair, Δ = log *I*^*PM *^- log *I*^*MM*^, and subsequently fitted the NNN-sensitivity model of rank *r *= 3 to the intensity data of the three data sets given in Table 2.

**Table 2:** Chip characteristics of selected data sets studied.

Data Set	HG133A_S	Mouse	ENCODE	HG133P_Z	HG133A_Z
GEO^*a*^	GSE1133	GSE12545	GSE6292	GSE3061	GSE3061
Chip type	HG U133A	MG 430 2.0	Human Tiling	HG U133plus2	HG U133A
# probes × 10^6 *b*^	≈ 0.5	≈ 1.0	≈ 1.5	≈ 1.2	≈ 0.5
# probesets^*b*^	22,300	45,101	300, 000^*c*^	54,675	22,300
% absent^*d*^	61.9%	63.1%	94.8%	54.9%	42.8
⟨ log *L*^*N*^⟩_*chip*_^*e*^	2.0	2.3	1.1	1.94	2.09
log *I*_*max*_^*e*^	4.48	4.71	3.45	4.32	4.45
%(*GGG*)_1 _probes^*f*^	2%	1.9%	2%	2%	2%
%(*GGG*)_1 _probesets*^f^*	20%	19%	-	20%	20%

^*a *^Gene expression omnibus (GEO) accession number

^*b *^number of probes and of probesets per array

^*c *^pseudo sets are assembled using five consecutive probes

^*d *^percentage of absent probes per array

^*e *^mean value of the logged non specific background intensity and logged saturation intensity

^*f *^percentage of probes containing the (*GGG*)_1_-motif and of probe sets containing at minimum one of these probes

**Figure 7:**
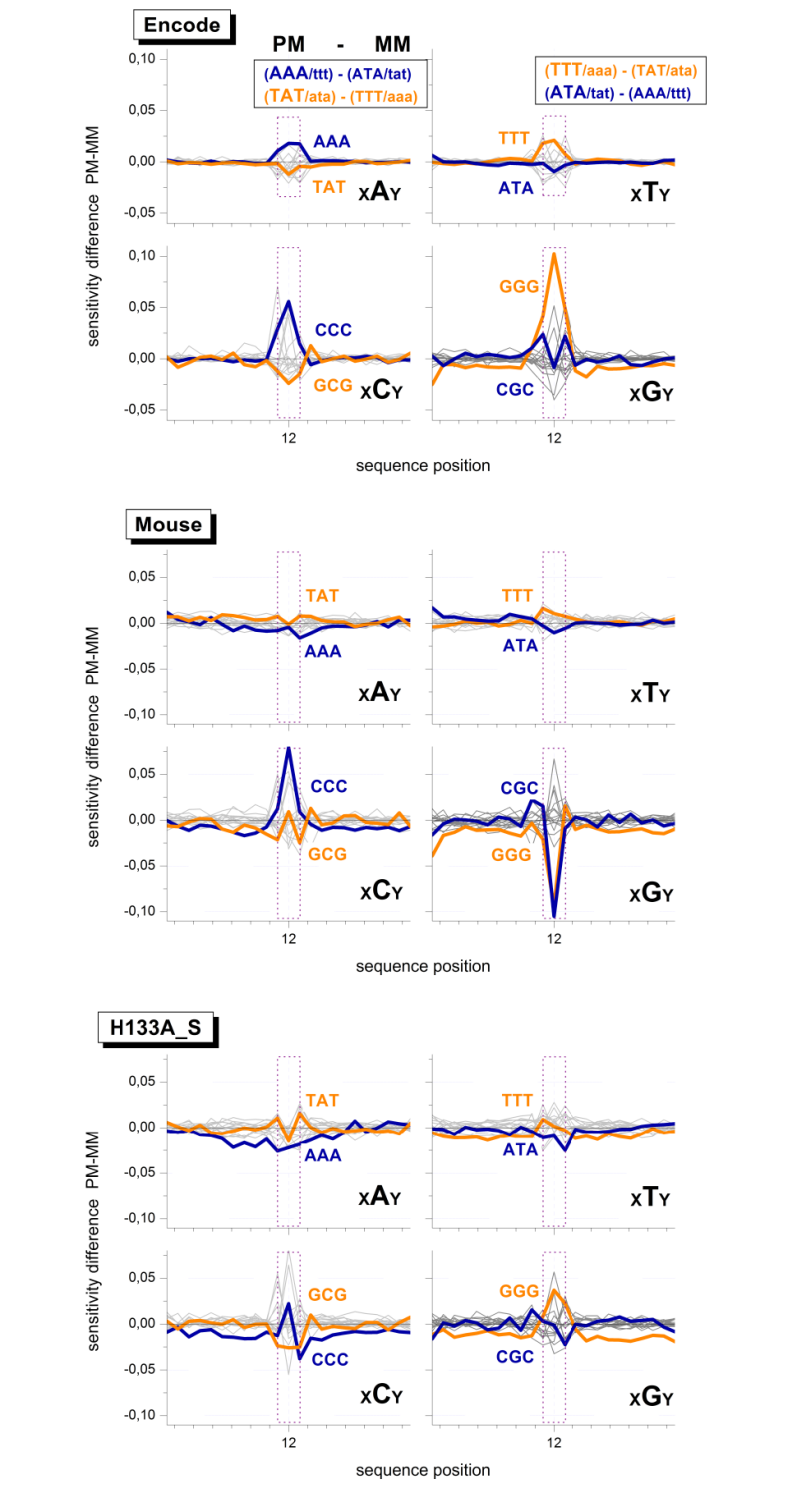
**Sensitivity difference profiles obtained by fitting the positional dependent NNN model to the logged intensity difference of each probe pair, Δ = log *I*^*PM *^- log *I*^*MM*^**. The profiles are sorted according to the central base in the NNN-terms of the PM probes, xBy with B,x,y = A,C,G,T. Only profiles of triple degenerated homo-motifs and the respective 'mirror' motifs with complementary center base are highlighted by colored thick lines. The upper panel gives the respective base pairings in the duplexes of the PM and MM (upper and lower case letters refer to the probe and the target respectively). The dotted rectangles refer to the middle tripe (sequence position *k *= 11 ... 13). The triple terms within this range are different for PM- and MM-probes. Their amplitudes refer to the respective swap of the middle base at *k *= 13 in the sequence of the MM-probes. Note the symmetries of the obtained profiles for complementary degenerated triples. The PM and MM probe sequences are identical outside the middle range. The absence of large amplitudes indicates that the intensities of both, PM and MM probes, similarly respond to sequence effects.

The obtained terms characterize subtle intensity differences between both probe types in a motif- and position-dependent way. Their amplitudes virtually vanish for *k *< 11 and 13 <*k *(Fig. 7). This result seems trivial because the sequences of PM and MM probes are identical at these positions. It clearly indicates, however, that the (*GGG*)_1_- and poly-G effects apply to the PM and MM probes as well.

The NNN-sensitivity data markedly deviate from the baseline at positions *k *= 11 ... 13 at which the triple motifs diverge between the PM- and MM-probes owing to the swapped middle base (in Fig. 7 this range is indicated by the dotted vertical lines). Here we focus our discussion to triple degenerated homo-motifs in the middle of the PM- (or MM-) sequence which combine with motifs of broken degeneracy in the respective paired MM- (or PM-) sequence. For example, (*GGG*)_11 _combines with (*GGC*)_11_, (*GGG*)_12 _with (*GCG*)_12 _and (*GGG*)_13 _with (*CGG*)_13_. The calculated sensitivity amplitudes consequently characterize the logged intensity difference due to both motifs.

Fig. 7 sorts the profiles with respect to the central base *B *of the middle triples in the PM sequence, xBy with *B, x, y *= *A, C, G, T*. The complete base pairings in the triple motifs are given in the figure. Base pairings in DNA/DNA duplexes are symmetrical with respect to bond reversal [26]. One expects therefore a central symmetrical pattern for the profiles of degenerated triples and the triples with swapped central base, e.g. AAA versus ATA and TTT versus TAT. The obtained sensitivity-profiles indeed show this symmetrical pattern. One expects also equal amplitudes for complementary homo-motifs, e.g. AAA and TTT. The observed effect however ranks according to *AAA *≈ *T T T *<*CCC *<*GGG*. The slightly larger peak of (*GGG*)_12 _compared with (*CCC*)_12 _indicates the poly-G effect along the sequence.

The mouse and HG133A_S data sets refer to DNA/RNA hybridizations. The chemical asymmetry of base-pairings between the DNA probes and RNA targets (see, e.g., [27,28]) explains the slightly modified pattern of the obtained triple motifs compared with that of the ENCODE data set. Particularly, one gets for the mouse data set *GGG *≪ *AAA *<*T T T *≪ *CCC *which is compatible with solution data (see also below). It therefore provides no indication of the poly-G effect. In contrast, in the HG133A_S data one observes the reversed relation for guanines and cytosines, *GGG *> *CCC*, which indicates a slightly larger intensity contribution of degenerated runs of guanines.

In summary, the joint analysis of the PM- and MM-intensities shows that both probe types are affected by the poly-G and (*GGG*)_1_-effect to a similar extent. It also reveals a relatively large intensity contribution of poly-G motifs in the middle of the sequence in some cases. The amplitude of this effects is however relatively small compared with the (*GGG*)_1_-effect.

### Specific and non-specific hybridization

Our analysis so far mainly use the positional sensitivity profiles of non-specifically hybridized PM probes and of the logged PM-MM difference. Selected profiles due to specific hybridization revealed a decreased sensitivity level of runs of degenerated guanines and, in particular, of the (*GGG*)_1_-motif (see the right part of Fig. 2 for the mouse data set, the specific profiles of the other data sets analyzed are given in [Additional file 1]). This result suggests that the (*GGG*)_1_-effect is only weakly or even not at all associated with specific hybridization.

It should be taken into account, however, that the specific sensitivity profiles are relatively uncertain owing to incomplete correction for parasitic effects such as saturation of the probe spots and bulk hybridization which deform the shape of the profiles and shift their level against each other [1,29,30]. Moreover, the number of probes in the sub-ensembles of probes used for calculating the specific profiles are typically much smaller than that of the non-specific probes. In addition, the specific sub-ensemble of probes is typically contaminated with contributions due to non-specific hybridization. All these factors give rise to relatively noisy profiles which still reflect properties of non-specific hybridization.

**Figure 8:**
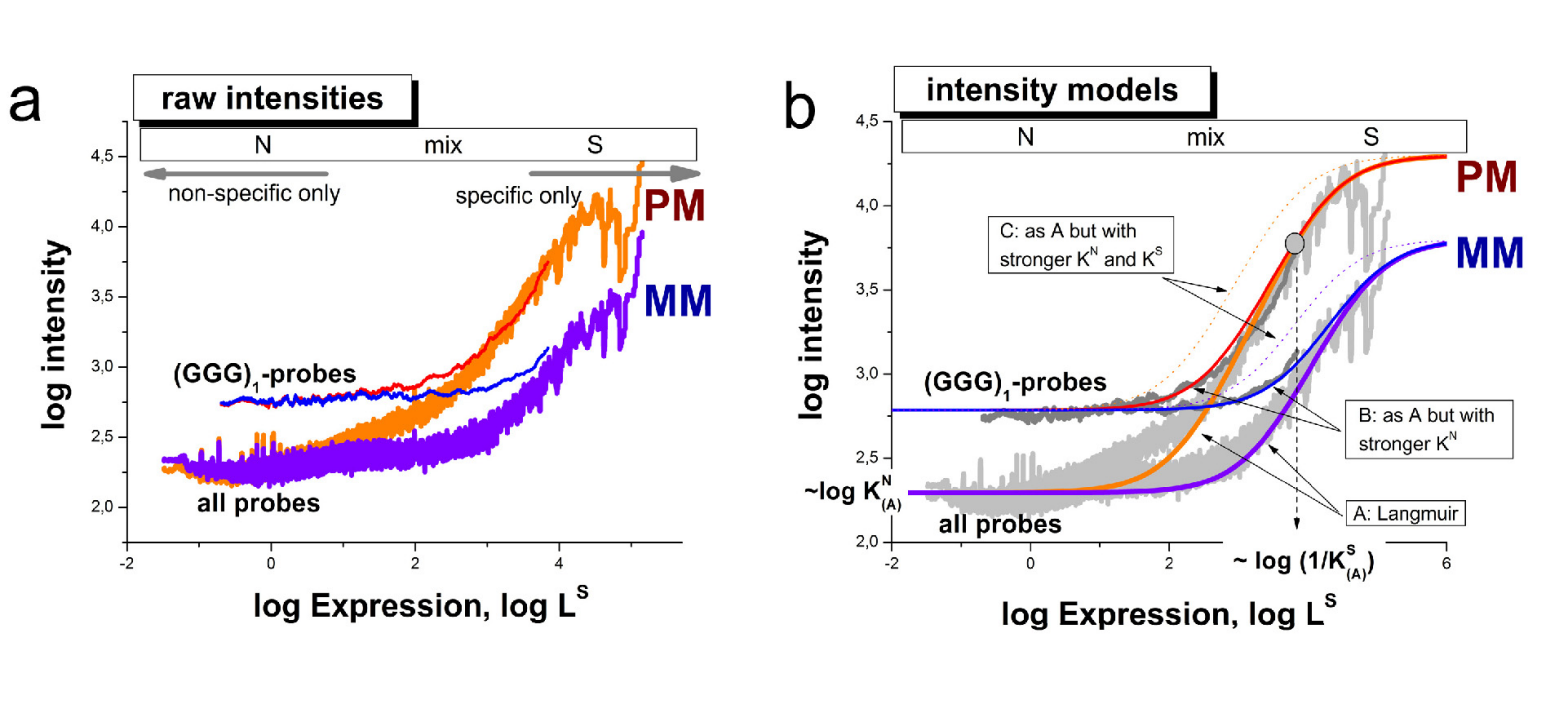
**Hybridization isotherms of the mouse data set: The isotherms in panel a were calculated by plotting the probe intensities as a function of their expression value which is directly related to the concentration of specific transcripts, *L*^*S *^∝ [*S*]**. The data were subsequently smoothed over a moving window of 1000 probe intensities. The isotherms were calculated using either all PM- or MM-probe data of the chip or, alternatively, the sub-ensemble of probes containing the (*GGG*)_1_-motif, i.e. a run of three consecutive guanines starting at the first sequence position. The horizontal bar in the upper part of the figure assigns the hybridization ranges (N, mix and S) which are described in the text. The arrows indicate the regions which are dominated either by specific or non-specific hybridization. Panel b shows theoretical isotherms which were calculated using Eq. (2) assuming three scenarios: (A) the reference situation describing the behavior of all probes; (B) stronger non-specific binding compared with A and; (C) stronger non-specific and specific binding compared with A (see also the text for details). Note that the intensity level in the N-range is directly related to *K*^*N*^, the mean binding constant of non-specific hybridization, whereas the position of the inflection point halfway between the N- and asymptotic saturation levels is inversely related to 1/*K*^*S*^, the mean binding constant of specific hybridization as indicated in the figure. The experimental data are compatible with stronger non-specific binding and invariant specific binding to (*GGG*)_1_-motifs compared with the respective main level of the binding strength of the array (scenario B). The MM-probes virtually behave like weak-affine PM probes with respect to specific binding.

We therefore apply a different approach to answer the question whether the (*GGG*)_1_-effect extends also to specific hybridization or not. Part a of Fig. 8 plots the smoothed probe intensities of the mouse data set as a function of the expression degree which was calculated using the hook method [31,32]. This calibration approach inverts the two-species Langmuir hybridization isotherm and estimates the linearized intensity-equivalent due to specific hybridization *L*^*S *^(see Eq. (2)) using the respective raw intensity values. The graphs in Fig. 8 thus characterize the mean dependence of the intensity as a function of the specific transcript concentration [S] which is directly related to *L*^*S*^. These isotherms roughly divide into the N-range which is dominated by non-specific hybridization at small abscissa values; into the S-range in which the intensity is dominated by specific hybridization at large abscissa values and into the mix-range in-between, in which both, specific and non-specific hybridization significantly contribute to the observed intensity (see also Fig. 8 for assignment).

Fig. 8 shows two different isotherms for the PM and MM probes each. One was calculated by averaging over all PM- (or MM) probes of the chip and the other one by selecting the respective sub-ensembles of probes containing the (*GGG*)_1_-motif. In the N-range, the intensity level of the (*GGG*)_1_-containing probes is clearly larger compared with that of all probes. The respective log-intensity increment of about 0.5 roughly agrees with the sensitivity amplitude of the (*GGG*)_1_-motifs *σ*_1_(*GGG*) ≈ 0.4 (see Fig. 2). The difference between both types of isotherms, however, progressively decreases with increasing expression degree and virtually vanishes in the S-range.

In panel b of Fig. 8 we plot theoretical isotherms calculated using Eq. (2) with the substitution *L_p_*→ *L *= *I*^*max*^·(*K*^*S *^[*S*] + *K*^*N *^[*N *]) as a function of the specific transcript concentration [*S*]. Three scenarios, (A) - (C), are considered to interpret the experimental data: (A) The 'reference' case with a parameter set which was chosen to fit the mean isotherms of the array averaged over all PM- or MM-probes; and scenarios (B) and (C) which aim at reproducing the behavior of the (*GGG*)_1_-subensemble. Particularly, in scenario (B) only the value of the non-specific binding constant is increased compared with the reference case (A) according to  whereas the value of the specific binding constant remained unchanged  = . In scenario (C) also the value of the specific binding constant is increased by the same factor as *K*^*N *^in case (B), i.e.  and .

Comparison of the theoretical and experimental curves clearly reveals that the intensity increment of the (*GGG*)_1_-containing subensemble is readily described by the second case (B) which only assumes the stronger non-specific binding of the probes. Case (C) assumes also an increased specific binding. It clearly fails descibing the data: The inflection point of the calculated isotherms shifts to smaller abscissa values whereas that of the experimental isotherms remains roughly at the same position.

Hence, comparison between measured and calculated isotherms provides no indication that specific hybridization contributes to the (*GGG*)_1_-effect to a similar extent as non-specific binding. Instead they show that the (*GGG*)_1_-effect is mainly associated with non-specific hybridization.

The isotherms of the MM-probes are shown in Fig. 8 together with the isotherms of the PM-probes. Both probe types are equally affected by non-specific hybridization on the average in both considered probe ensembles. Particularly, the (*GGG*)_1_-motif increases the intensity level of the MM-probes in the N-range to the same extent as that of the PM-probes. The slight shift of the mix- and S-ranges of the MM-probes towards larger expression values is caused by the weaker specific binding of the MM due to their swapped middles base which mismatches the target sequence. Hence, the MM-probes virtually behave like weak-affine PM-probes with respect to specific hybridization. This difference also implies that the mean saturation intensity of the MM-probes *I*^*max *^is smaller than that of the PM-probes owing to post-hybridization washing [33,34]. The calculated isotherms of the MM-probes clearly show that specific binding is virtually not affected by the (*GGG*)_1_-motif by the same arguments as for the PM-probes.

### The NN+GGG correction

Our analysis shows that the quality of fit of sequence models is heterogeneous with respect to the selected motifs and their position along the probe sequence. The positional dependent NN model well describes most sequence-dependent intensity effects due to non-specific hybridization with the exception of motifs of three or more consecutive guanines. Higher order models of rank *r *= 3 or 4 are able to successfully remove the associated sequence bias, however they are expensive to compute. Minimization of the linear regression model Eq. (7) provides a system of (4*r *- 1)(25*r *+ 1) linear equations, the solution of which requires a runtime in the order of *O*(#*p*(4*r*)^2^). In practice, profiles with rank up to *r *= 2 can be computed in minutes per array on a standard personal computer whereas models of rank *r *= 3 and 4 run hours or even days, respectively.

We therefore developed a hybrid-rank model based on the positional dependent nearest neighbor approach plus additional higher order contributions for selected 'critical' motifs such as (*GGG*)_1 _which applies to the intensity components due to non-specific binding (see previous subsection). The algorithm fits the NN-model of rank r = 2 to all probes which do not contain the critical poly-G motifs in their sequence. The intensities of probes which contain such motifs are separately fit to a NNN model of rank *r *= 3 which only considers triple-G motifs at all possible sequence positions. In general, this approach can be modified to apply to other special motifs.

**Figure 9:**
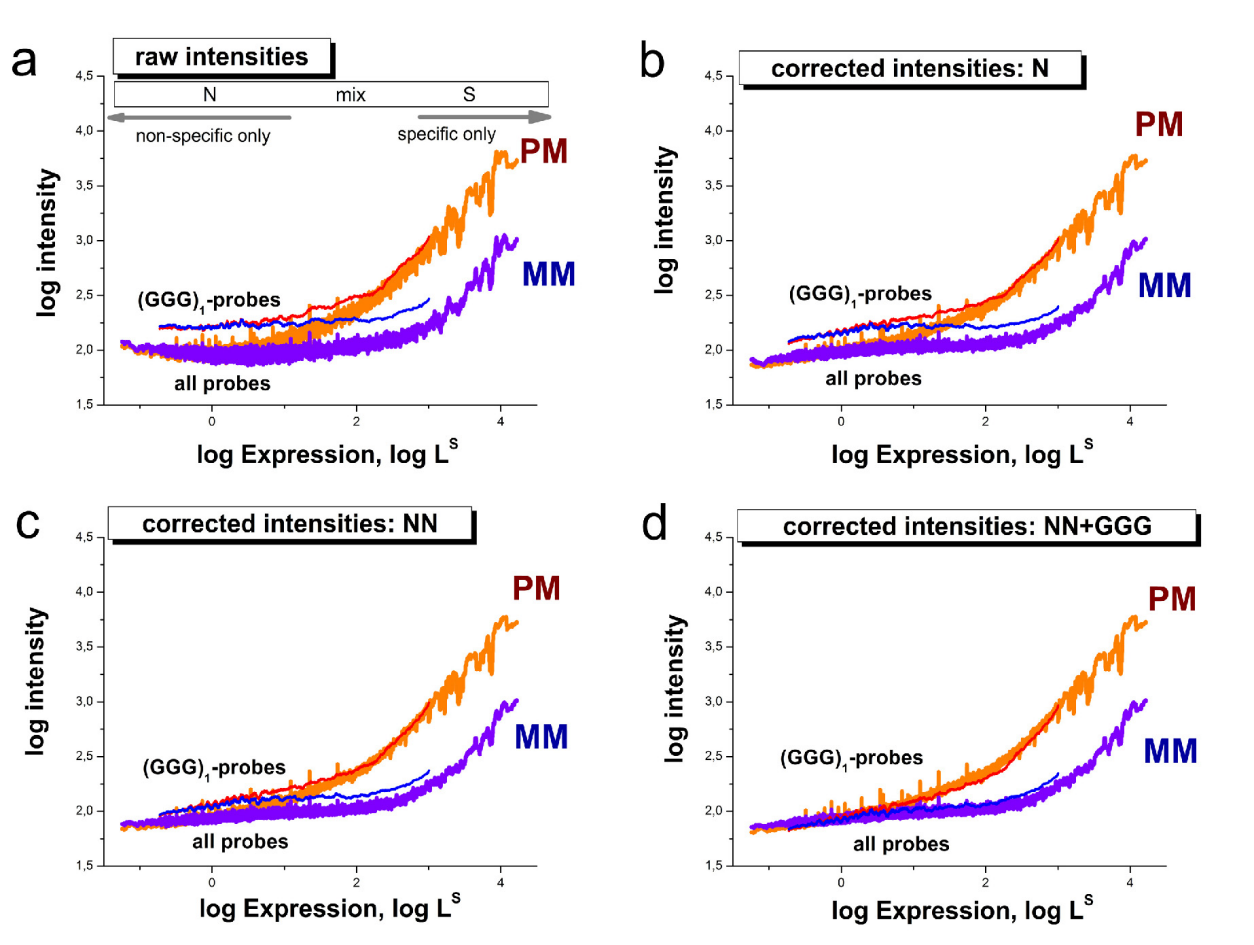
**Correction of microarray intensity data using models of rank *r *= 1, 2 and the hybrid rank model NN+GGG for the non-specifically hybridized probes of the mouse data set**. Specific hybridization is corrected using the NN-model in all cases. The figure shows the averaged intensity as a function of expression as in Fig. 8. The systematic bias of probes containing the (*GGG*)_1_-motif progressively decreases with increasing rank of the model and it virtually vanishes for the NN+GGG model. Correction using the NNN model provides a plot which is virtually indistinguishable from that of the NN+GGG model (not shown).

Fig. 9 compares the performance of the hybrid rank correction with that of the N and NN models using the same type of representation as in Fig. 8 above. It clearly shows that the latter two models only insufficiently correct the (*GGG*)_1_-effect as expected. On the other hand, the systematic bias of the (*GGG*)_1_-containing probes in the non-specific hybridization range almost completely vanishes after applying the NN+GGG correction to the non-specifically hybridized probes using the algorithm described in the Methods subsection below.

**Figure 10:**
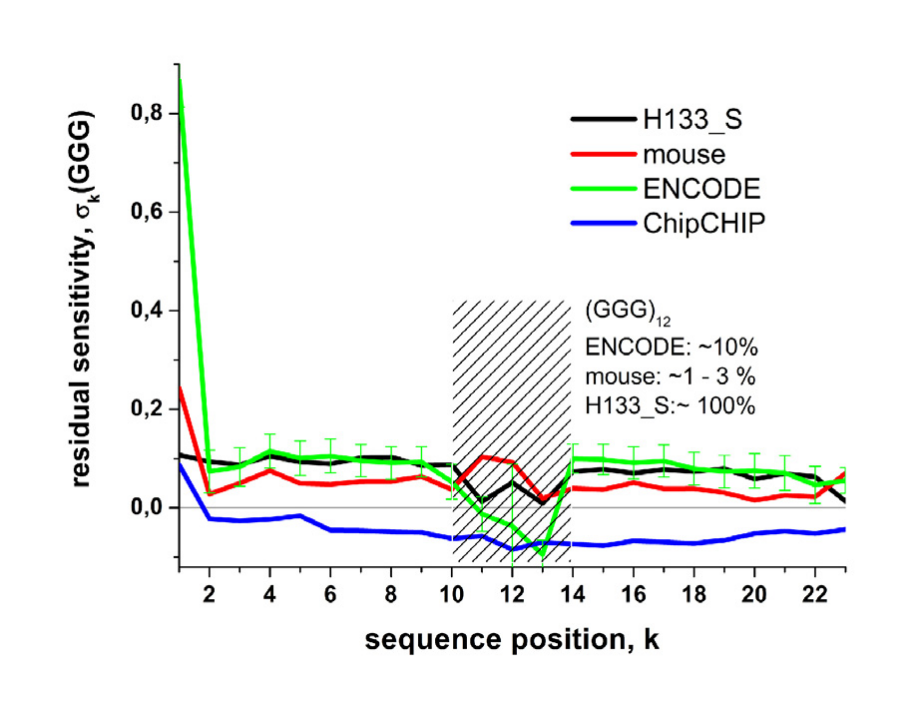
**Positional dependent residual sensitivity profiles of triple-G motifs**. The data clearly reveal the poly-G and the strong (*GGG*)_1_-effect of the mouse and ENCODE data sets. The hatched region refers to sequence positions with very small numbers of probes containing the (*GGG*)_12_-motif printed on the mouse and ENCODE arrays (see [Additional file 1]). Interestingly, ChipChIP applications of the ENCODE arrays give rise to negative residual GGG-sensitivity values for most of the sequence positions.

The residual profiles of the triple-G motifs of the three data sets used in Fig. 7 are shown in Fig. 10. They clearly reveal the strong intensity excess at position *k *= 1 due to the (*GGG*)_1_-effect (mouse and ENCODE data sets). The mean level of the poly-G effect affecting the remaining sequence positions is about *σ*_*k*_(*GGG*) ≈ 0.1 for these chips. This excess sensitivity value refers to an intensity bias of 10^0.1 ^≈ 1.25 compared with the NN-model. Interestingly, hybridizations of ENCODE arrays using the ChipChIP technique indicate a negative GGG-level throughout the sequence for *k *> 1. It indicates an average intensity bias in the opposite direction of about 10^-0.07 ^≈ 0.85.

We argued above that the ChipChIP targets lack G-rich primer fragments which otherwise cause the strong intensity bias due their involvement into G-stack formation on expression arrays. Their absence would explain a tiny or even zero but not a negative amplitude-level of the triple-G excess sensitivity. A similar negative sensitivity effect of poly-G motifs has been found recently for SNP GeneChip arrays [10]. These arrays also use genomic DNA for hybridization after amplification via ligation and not via T7 priming [35]. This 'dim' effect has been attributed to G-stack formation of four neighboring probes in agreement with previous assumptions [9,36]. Such probe quadruplexes reduce the amount of free probe oligomers available for the binding of specific and non-specific targets. This trend then decreases the intensity of the respective probe spots because only targets are labeled with optical markers.

In summary, the NN+GGG hybrid-rank model properly corrects the intensity bias associated with probes which contain poly-G motifs. In addition, the obtained excess GGG-profiles provide further insights into the amplitude of the effects due to degenerated guanines in different hybridizations. It changes sign and switches from positive to negative values for hybridizations which use different amplification protocols.

## Discussion

### Preprocessing of microarray intensity data

Calibration of microarray measurements aims at removing systematic biases from the probe-level intensity data to get expression estimates which linearly correlate with the transcript abundance in the studied samples. The performance of different preprocessing algorithms to correct intensity data for the (*GGG*)_1_-effect are illustrated in Fig. 1b by means of boxplots which roughly characterize the distribution of the expression values in terms of their median and interquartile range. The results revealed that the strong intensity effect is not removed from the expression data after standard preprocessing using several popular methods.

**Figure 11:**
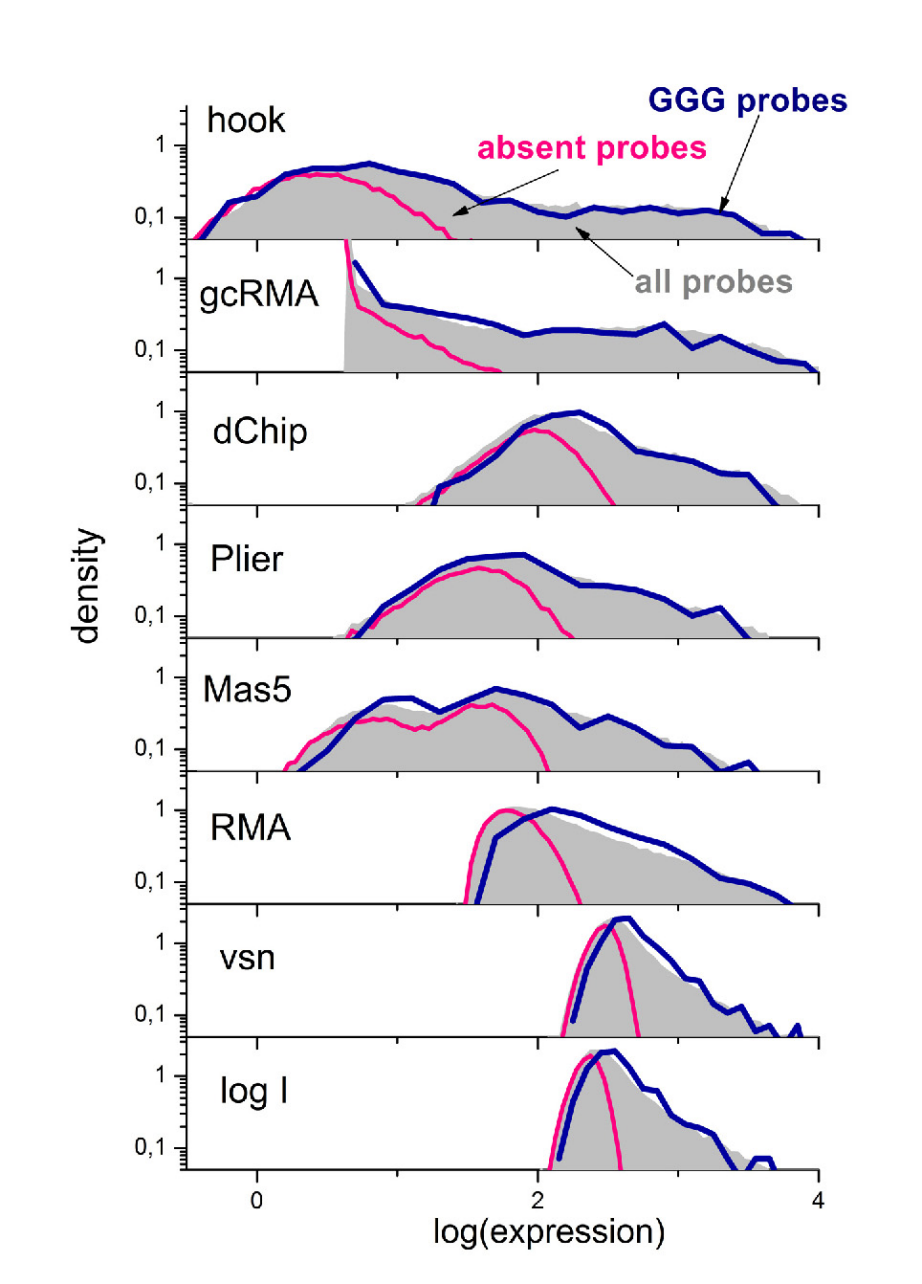
**The distribution of expression measures obtained from intensity data shown in Fig. 1 and various preprocessing methods**. The whole density distributions reveal subtle differences produced by the different methods. The distributions are computed separately for all probe sets (45,100) and for probe sets with at least two probes containing a (*GGG*)_1_-motif (836, i.e. 2% of the total number) and for absent probe sets hybridized exclusively nonspecifically (45% of all probes). The multichip methods (RMA, gcRMA, dChip, vsn, Plier) are applied by computing intensity data of 5 arrays from the respective experimental series. 'log I' denotes the distributions of raw intensity data. The distributions of expression measures of probe sets containing (*GGG*)_1 _probes for RMA, gcRMA and to a less degree for MAS5 and dChip are systematically shifted to the right compared with the distribution of all probe sets. These methods are partly unable to correct expression values for the (*GGG*)_1_-bias whereas hook and Plier remove the bias.

To get further insights we plot the density distributions of the preprocessed expression values of all 45,100 probe sets of the mouse data set and of the sub-ensemble of 836 probe sets containing at minimum two probes with a (*GGG*)_1_-motif (Fig. 11). The results indicate the systematic shift of the (*GGG*)_1 _sub-ensemble towards larger expression values in decreasing order for the preprocessing methods vsn [2], RMA [3] and gcRMA [4]. Note that vsn and RMA use global baseline-corrections for non-specific hybridzation which subtracts one common background value from all probe intensities of a selected microarray. Clearly these approaches fail to describe the probe specifics of the (*GGG*)_1_-motif giving rise to a strong bias due to improper background correction.

Fig. 11 also shows the distribution of the sub-ensemble of 'absent' probe sets (49% of all probe sets) which have been identified using the hook method [32]. Comparison with the other distributions reveals that the amplitude of the (*GGG*)_1_-bias decreases with increasing expression value. However, it affects not only the range of non-specific background but extends to probe sets with a significant contribution of specific hybridization. These signals are potentially used in downstream expression analysis. The right tail of the distribution is dominated by specific hybridization which has been shown to remain virtually unaffected by the (*GGG*)_1_-effect.

The preprocessing methods dChip [5], gcRMA, MAS5 [6], Plier [7] and hook [32] apply probe-specific baseline correction algorithms which estimate an individual background value for each probe. The obtained distributions significantly widen, and extend towards smaller expression values offering a larger dynamic range of the obtained expression estimates. The detailed inspection of the density distributions however also reveals a small (*GGG*)_1_-bias in the left part of the distributions obtained by MAS5, dChip and gcRMA which is dominated by non-specific hybridization. The version of gcRMA used applies a positional dependent sequence correction of rank *r *= 1 similar to ours (Eq. (4)) which is obviously insufficient to account for the (*GGG*)_1_-effect. MAS5, dChip and also Plier explicitly use the intensities of the MM probes to estimate the non-specific background of the PM signals. PM- and MM-probes are both affected by the poly-G motifs to a similar extent which enables its effective correction by combining PM- and MM-data. Finally, hook and Plier almost completely remove the (*GGG*)_1_-bias from the data over the whole width of the distributions.

**Figure 12:**
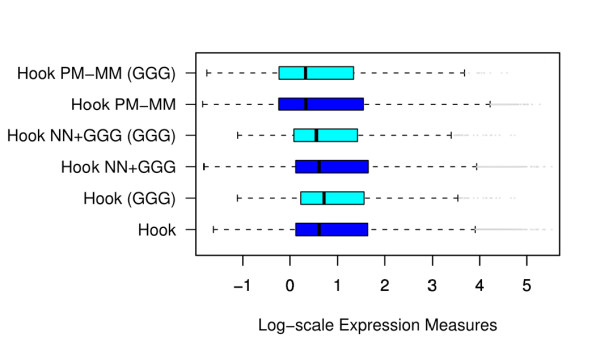
**Boxplots of expression measures of all probe sets and of probe sets containing (*GGG*)_1_-motifs obtained from the intensity data shown in Fig. 1 (mouse data set) using different versions of the hook method **[32]: The PMonly approach in combination with the NN sensitivity model (hook) only insufficiently corrects the (*GGG*)_1_-effect. The combinations of PMonly and NN+GGG (hook NN+GGG) and PM-MM difference and NN (hook PM-MM) provide good results.

The shown hook distribution refers to the PMonly variant of the method [32] with implemented NN+GGG sensitivity correction which neglects the intensity information of the MM probes. The so-called 'PM-MM' variant of the method also processes the intensities of the MM probes. Fig. 12 compares the median position and the width of the distributions of expression values of different versions of the hook method in terms of boxplots. The PMonly and PM-MM standard versions apply the positional dependent NN-model. The PMonly variant only insufficiently removes the (*GGG*)_1_-bias whereas the PM-MM method provides acceptable results. This finding confirms our suggestion that the explicit consideration of the MM-intensities enables better correction of the sequence bias. The NN+GGG sensitivity model provides acceptable correction also for the PMonly version of the method.

**Figure 13:**
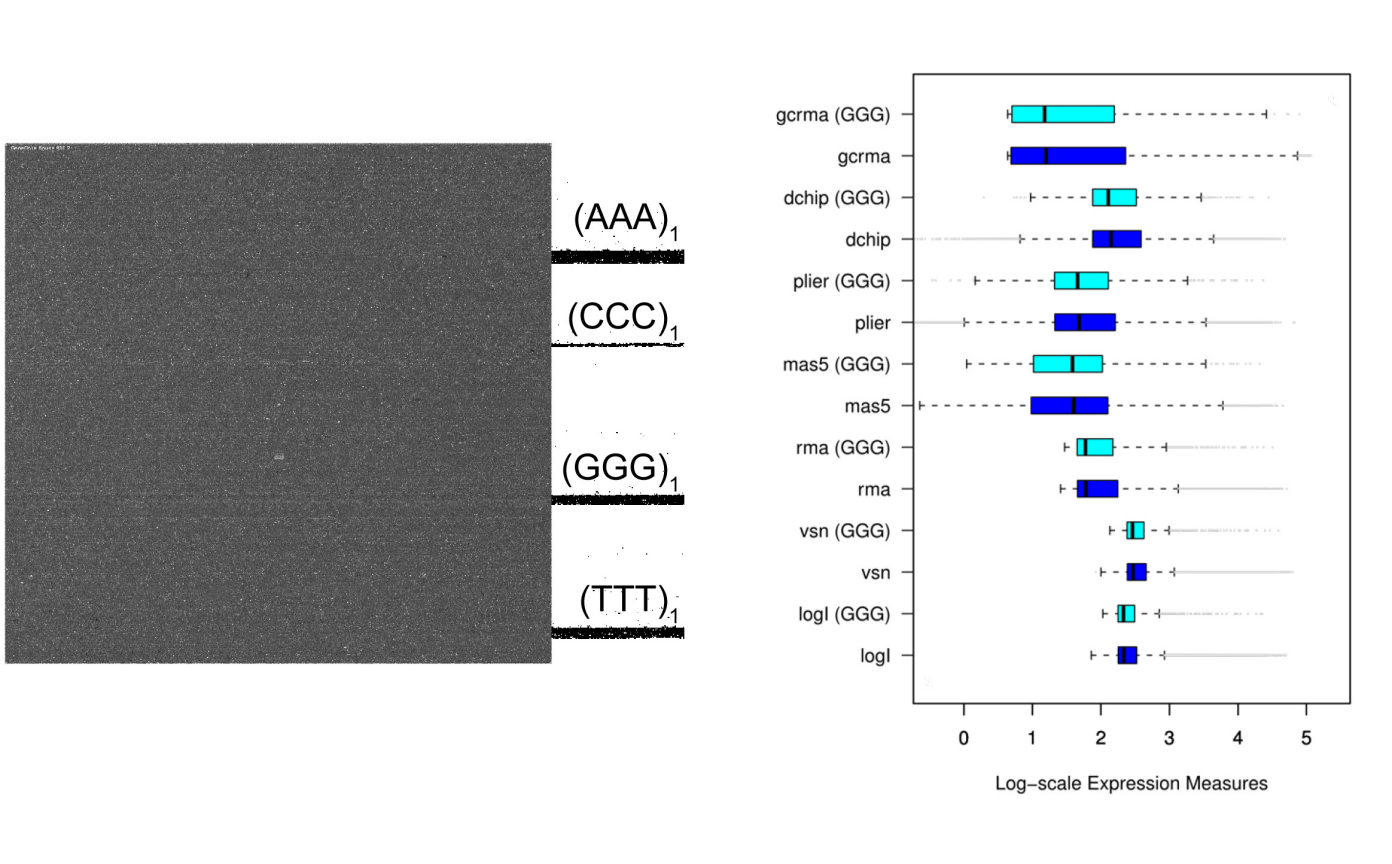
**The figure shows the same data as in Fig. 1 after sensitivity correction using the NN+GGG model**. (a) Pseudo image of the chip calculated from the cel-file of corrected intensities. The bright stripes seen in Fig. 1a disappeared. (b) Boxplots of expression measures obtained from the pre-corrected intensities. The GGG-bias essentially vanishes after correction (compare with Fig. 1).

Fig. 13 reproduces Fig. 1 for corrected intensity values using the NN+GGG model. Panel a shows a pseudo-image of the array using the CEL-file of corrected intensities. The bright stripes due to the (*GGG*)_1 _probes evident in Fig. 1a clearly disappeared. Panel b illustrates the performance of different preprocessing methods with respect to the (*GGG*)_1_-bias after applied correction. The boxplots clearly show that our correction effectively removes the (*GGG*)_1_-effect from the resulting expression values. In summary, most of established preprocessing methods only inadequately calibrate raw intensity data for strong sequence effects of the non-specific background contribution. Methods which explicitly process suitable reference probes, such as the MM, perform better than PMonly methods. Pre-correction of the intensity data using the NN+GGG sensitivity model removes the bias due to degenerated guanines from the data.

### Sequence-specific intensity corrections

The correction for sequence-specific intensity effects is a crucial step which largely affects the performance of the preprocessing of microarray data. It applies to specific hybridization ('affinity' correction) and also to non-specific hybridization as well (correction for the chemical background). Numerous sequence models have been developed for microarray analysis so far. They can be roughly divided into the following four classes:

(i) 'Fully' physical, Δ*G *based approaches (here Δ*G *symbolizes the change of the free energy upon probe/target binding) [14,30,37-44]: These models explicitly and in-detail consider different processes which potentially affect probe hybridization such as probe/target duplexing including their zippering, bulk dimerization of the targets or folding of target and probe in terms of effective reaction constants or statistical thermodynamics. Elementary interactions are described on the level of base pairings using stacking free energy parameters which have been estimated in independent dimerization experiments of oligonucleotides in solution [10,26,45]. Such models helped to improve our basic understanding of the functioning of microarrays and also to judge the relevance of different contributions to the observed probe intensities. These approaches often apply special fitting approaches and/or idealized assumptions to describe intensity data of selected microarray experiments (for example spiked-in data sets). Often, the used tools and algorithms however fail in practical microarray analysis because particular factors significantly affecting the performance of chip measurements are either considered in a simplified fashion or even neglected. For example, the lack of knowledge about the exact length, full sequence and concentration of the targets circumvents the detailed estimation of their folding and duplexing products. On the other hand, these 'physical' models clearly showed that microarray hybridization is in agreement with elementary physical rules of interacting probes and targets, which however take place in a complex environment owing to the attachment of probes to the chip surface and the heterogeneous composition of the target solution. The latter conclusion was also supported by the results of reverse top-down studies which extract interaction parameters on the level of base pairings from microarray intensity data. For example, the resulting intensity-based NN parameters in most cases correlate well with the respective stacking free energies of independent solution experiments [10,14,27,40].

**Figure 14:**
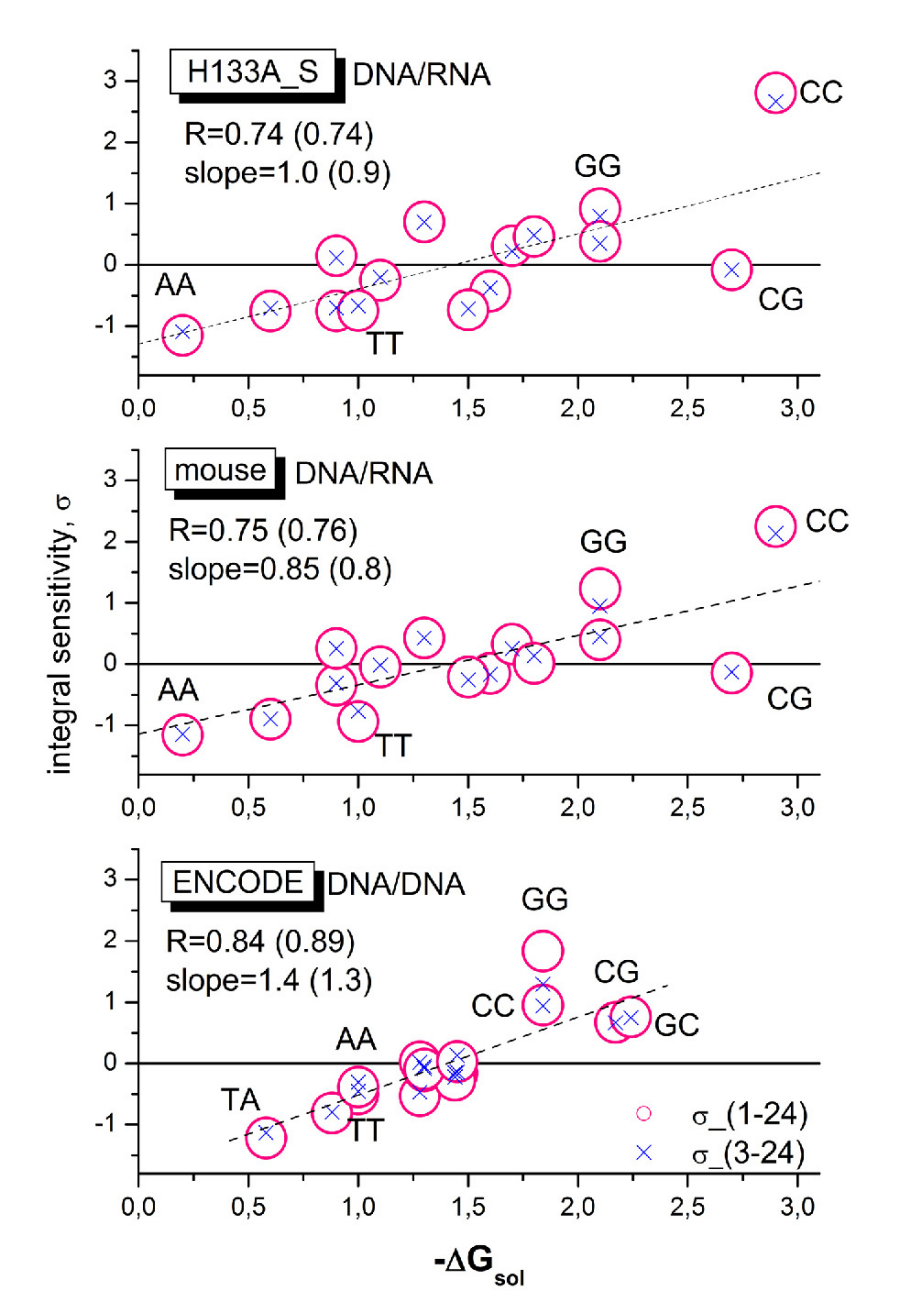
**Correlation plots between the integral sensitivity of the positional dependent NN-model and solution free energies of DNA/DNA- and DNA/RNA-hybridizations taken from **[26,46]** and **[28]**, respectively**. The three panels refer to DNA/DNA (ENCODE) and DNA/RNA (mouse, HG133A_S) hybridizations. The integral sensitivities are calculated using either all sequence positions (circles) or positions 3 - 24 (crosses). The latter data are normalized using the normalization factor 24/21 for direct comparison with the former data. Regression lines are shown for the latter data. The regression coefficients (R) and the slopes are given in the figure. The values in parentheses refer to the reduced sum. Note that the integral sensitivities of GG nearest neighbor motifs clearly decrease if one neglects the first two sequence positions. The effect on the regression remains however small. Selected NN motifs are assigned in the figure.

The present study confirms this result (Fig. 14). In particular, we calculated the sum of all terms of the NN profiles over all 24 sequence positions for the selected data sets to obtain positional independent mean sensitivity estimates. The obtained integral NN terms were correlated with the respective nearest-neighbor free energies for DNA/DNA or DNA/RNA duplexes in solution which were taken from [26,46] and [28], respectively. The microarray sensitivities well correlate with the solution free energies (regression coefficients of *R *> 0.7). To judge the amplitude of the (*GGG*)_1_-effect on the integral NN terms we calculated a second data set which omits the first three sequence positions in each sum for the integral NN terms (see the crosses in Fig. 14). Only the values of the GG terms reduce notably in the mouse and ENCODE data sets accompanied by a small improvement of the respective fits.

The latter result shows that global parameter estimates can mask special intensity effects associated with selected sequence motifs such as runs of guanines, which results in the poor modeling of the intensities of probes containing these motifs.

(ii) Positional dependent intensity models with freely adjustable parameters in analogy to the approach used in this study: This class of models was independently introduced by Mei et al. [11] and Naef and Magnasco [18] which originally use single base terms, rank *r*=1. Shortly after the method has been upgraded to NN-terms of rank *r*=2 [47] and successfully applied in different calibration algorithms for microarray data using either N- [19,48] or NN-models [8,32,49-51]. The parameters are estimated individually for each array. The model thus accounts for the specifics of each particular hybridization which potentially varies from chip to chip due to different levels of non-specific hybridization, bulk dimerization, washing and/or saturation. All these effect are shown to modify the respective parameter profiles [17,29]. The obtained parameters are therefore called effective affinities [17] or sensitivities [1,16] depending on the special experimental setup. Moreover, the model also enables to describe subtle differences between non-specific and specific hybridization on the level of base pairings, for example, due to the presence of defined mismatches in the probe/target duplexes [15,16,32]. The approach successfully applies to chips of different generations and types [31,48] and it can be combined with elements of model class (i), for example, to account for probe and target folding [49,51] or for special motifs and additional factors [11,48]. For example, the pioneering approach of Mei et al. [11] combines the positional dependent N-model with special correction terms for intramolecular hairpins and G-quadruplexes. The latter effect was separately assigned to runs of at least four guanines at the beginning, the middle and the end of the probes. Here we extended the model to positional dependent triple and quadruplex motifs of rank *r *= 3 and 4. Our analyses show that the NN-model well accounts for most of the sequence effects except special motifs such as runs of consecutive guanines. We also demonstrated the diagnostic power of this approach to detect subtle sequence effects in terms of position and motif.

(iii) Positional dependent approaches with common 'shape functions': This class of models is closely related to the previous class (ii). In contrast, it however factorizes the positional and motif dependent sensitivity profiles into two independent contributions namely into positional independent but motif specific 'energy' terms and into a positional dependent but motif independent 'shape'-function common for all motifs. This so-called PDNN (positional dependent nearest neighbor) model was originally introduced by Zhang et al. [52]. It is used with modifications in different algorithms and applications [14,53-55]. The common shape function of the PDNN model considerably reduces the number of adjustable sequence parameters by nearly one order of magnitude and consequently also the computational effort compared with the NN-model with motif specific profiles ((42 - 1) + 24 = 39 PDNN-parameters versus 361 NN-parameters, see Eq. (9)). It has however to be asked whether the common shape function adequately reflects the positional dependence of the individual NN-profiles? The inspection of the plots in Figs. 2 and 3 suggests, for example, that the shape of guanine-rich profiles strongly deviates from the shape of other motifs owing to the (*GGG*)_1_-effect. For a systematic evaluation we make use of the NN-model with adjustable positional sensitivities of class (ii) and compare all pairwise combinations of the 16 sensitivity profiles using a simple similarity metrics based on the least squares optimization of a scalable factor *a *and a shift-term *c*,(1)

**Figure 15:**
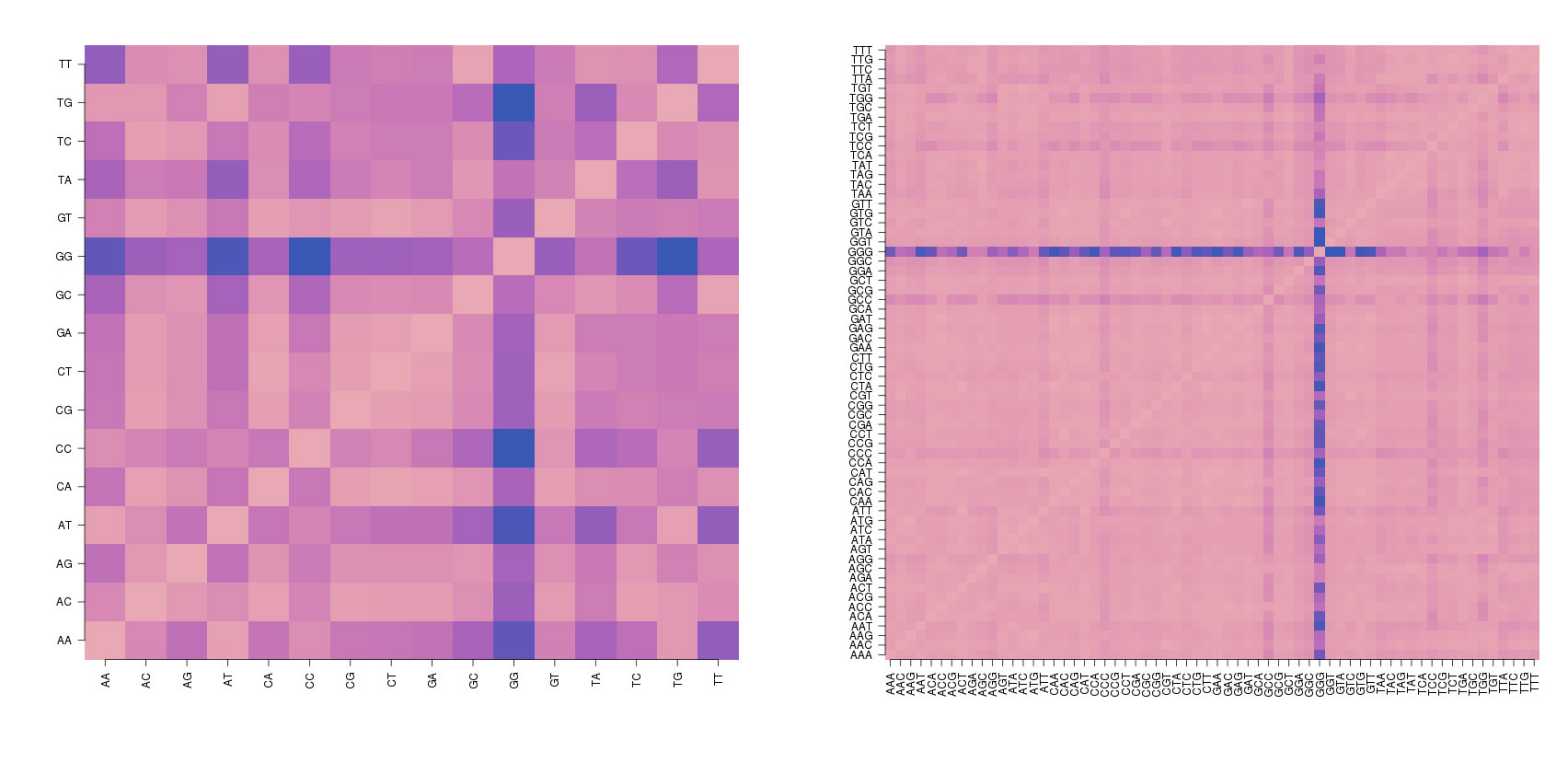
**Heatmaps of the similarity matrix *SI *(*b*_2_, *b*_2_) of the shapes of positional dependent sensitivity profiles of rank *r *= 2 (left panel) and 3 (right panel) of the mouse data set**. Pair-wise similarity is color-coded: dark spots indicate small similarity (see text).

Here *b*_1 _and *b*_2 _denote two selections from the 16 NN terms. The similarity matrix  indeed reveals that the profile of GG-sensitivities poorly matches the remaining profiles, except TT (Fig. 15, left panel). Bad or only moderate agreement is also observed between the profiles of other NN-motifs such as CC, AA and TT. The similarity matrix of the NNN-profiles of rank *r *= 3 reveals a similar picture with poor matches especially for GGG motifs and partly also for CCC and CCG (see right panel of Fig. 15). Hence, the assumption of a common shape fails for selected motifs.

iv) Multichip statistical models: These approaches decompose each probe intensity into independent factors due to probe and chip effects. The former factor is assumed to be invariant for each probe in a series of arrays and thus models the respective sequence-specific affinity of the probe. The latter factor is assumed to describe the expression index which usually varies between the chips. The relation between intensity and expression index is either linear (RMA, gcRMA, vsn, dchip, Plier) or hyperbolical assuming a Langmuir isotherm (Nlfit, [56]). The parameters are estimated by fitting the model to the intensities of a series of, at minimum, 5 - 10 arrays. The approach has the potential to correct the intensities for any probe effect because each probe is handled individually without explicitly processing its sequence in terms of a sequence model as in the alternative approaches of classes (i) - (iii). On the other hand, chip and probe effects are not independent in real situations due, e.g., to different levels of bulk dimerization and other effects (see above). More importantly, the probe-related affinity correction of the multichip methods in most cases applies to specific hybridization only whereas the non-specific background is corrected using simpler approaches such as global background (RMA, vsn) or N-profiles (gcRMA). Hence, the performance of the method largely depends on the type of background correction (see also the previous subsection).

Note that dChip and Nlfit assume a probe dependent background which partly removes the the (*GGG*)_1_-bias from the data (see the results for dChip in Fig. 11).

We conclude that hybrid models of class (ii) are conceptually best suited to account for special sequence effects in single-chip based calibration algorithms for microarrays which use a high number (> 10^5^) of short (length < 30 bp) oligonucleotide probes such as GeneChips. Here the large number of intensity values allows successful fitting of hundreds model parameters. Possibly, the performance of models of this class can be further improved using amendments taken from physical models of type (i), e.g. to consider the folding propensity of the targets and/or their length. The non-linear approach [56] offers an interesting option of models of class (iv) because it allows to apply adequate hybridization laws beyond the linear approximation in combination with sophisticated affinity corrections. Its multichip character, however, adds normalization tasks to consider variations between different hybridizations which might produce biased expression estimates [29]. Models of class (iii) must be complemented with special terms to account for special sequence effects deviating from the mean positional dependence of the array. With this amendment they represent an interesting choice for array-types using long oligonucleotide probes (length > 30 bp) because it requires fitting of a reduced number of positional parameters compared with models of class (ii).

## Conclusions

We analyzed the specifics of probe intensities on the level of short motifs of one to four adjacent nucleotides along the 25-meric probe sequence using positional dependent sensitivity models. The decomposition of the fit statistics into motif- and positional dependent contributions reveals that most of the motif-specific terms are adequately described using a nearest-neighbor model. In contrast, runs of degenerated guanines require explicit consideration of next nearest neighbor terms for adequate fitting. Longer runs of at minimum three consecutive guanines along the probe sequence and especially triple degenerated G at its solution end typically cause exceptionally large probe intensities on expression arrays. This intensity bias affects PM- and MM-probes to a similar extent. Our analysis clearly shows that this effect is associated with non-specific hybridization. Hence, the interpretation of the extraordinary strong signals of probes containing runs of degenerated guanines in terms of high expression levels of the respective genes seems not justified.

The (*GGG*)_1_-effect tends to increase gradually for microarrays of later GeneChip generations. It was detected for hybridizations which use DNA/RNA as well as DNA/DNA probe/target-chemistries. Different amplitudes of the guanine effect were found for hybridizations which apply different amplification protocols. In particular, the T7 amplification step for sample messenger RNA is associated with strong amplitudes of the guanine effect whereas amplification protocols for genomic DNA lacking T7 priming behave differently.

The origin of the very strong (*GGG*)_1_-effect is unknown. Its association with the T7 protocol however implies that the T7-amplified targets containing the G-rich primer fragments are prone to form mixed probe/target G-stacks via association with G-rich probe motifs. This effect in combination with the large concentration of G-rich targets in the hybridization solution then would facilitate their strong binding to G-rich probes resulting in their strong intensity. The absence of these G-rich target motifs in the ChipChIP hybridization possibly explains the much smaller intensity of the respective (*GGG*)_1_-probes compared with the ENCODE hybridizations. This hypothesis requires further verification using, e.g., methods developed in [25].

Established preprocessing methods only insufficiently remove the guanine bias from data. Methods which explicitly process the intensities of the MM probes as suitable references perform better than PMonly methods. We propose a positional dependent NN+GGG hybrid-rank model to correct the intensity bias associated with probes containing poly-G motifs. It can be applied prior to established preprocessing methods in a pre-correction step. The positional and motif dependent sensitivity models are conceptually best suited to account for special sequence effects in single-chip based calibration algorithms for microarrays which use a high number of short oligonucleotide probes such as GeneChips.

The structural rationale behind the guanine effects has been concordantly assigned to the propensity of degenerated G motifs to arrange into of stable stacks of guanine tetrads which bundle four oligonucleotide strands into molecular quadruplexes [9-13]. These structures potentially affect the efficiency of oligonucleotide synthesis and/or the hybridization of the probes to their target sequences accounting for the abnormal performance of G-runs on the array [10]. Upton et al. [9] suggested a mechanism which increases the intensity of poly-G containing probes via the local opening of regions in the vicinity of quadruplexes formed by adjacent probes.

Alternatively one can assume that G-rich probes form G-quadruplexes of different stoichiometry which involve either exclusively adjacent probe oligonucleotides or also non-specific targets containing longer runs of guanines. We suggest that T7 amplification contaminates the targets with G-rich primer fragments which drastically increase their propensity to form such mixed probe/target G-quadruplexes. This model predicts that the large concentration of G-rich targets in the hybridization solution gives rise to their strong binding to G-rich probes which finally causes their strong intensity. The absence of these G-rich motifs upon hybridization of genomic DNA then explains the much smaller intensity of the respective probes. The more detailed analysis of the structural rationale behind the guanine effect in terms of physical models is beyond the limits of this publication and will be given elsewhere.

## Methods

### Datasets

In this paper we investigate various data sets dealing with different generations and types of Affymetrix GeneChip arrays which were taken from the public Gene Expression Omnibus (GEO) data repository http://www.ncbi.nlm.nih.gov/geo/. The central examples are summarized in Table 2: (i) Human Genome HG U133A arrays taken from the 'HG133A_S' dataset were reanalyzed to verify the effect of G-stacks reported recently [13]. (ii) Identical human reference RNA was hybridized to both HG U133A and HG U133plus2 arrays in the ' HG133P_Z' and 'HG133A_Z' datasets [57]. The latter arrays offer smaller feature sizes (11 versus 18 μm) and a larger number of probesets (54.675 versus 22.300). All probes of the HG U133A are replicated on the the HG 133plus2 array allowing direct comparison of the signal response of identical probes upon hybridization with the same RNA. (iii) In the 'Mouse' dataset we analyzed Mouse Genome 430 arrays referring to the same generation as the HG U133plus2 array. (iv) The 'ENCODE'-dataset comprises human tiling arrays taken from the ENCODE-project [22]. This array-type not only contains a further increased number of probes but also uses different hybridization and labelling chemistries compared with the expression arrays of the other data sets. Particularly, cRNA-targets are replaced with cDNA targets and nucleotide-labelling throughout the sequence is changed into end-labelling. Arrays of the ENCODE type can also applied in ChipChIP experiments with altered amplification protocols to explore protein/DNA interactions. We included ChipChIP data to study the effect of the amplification protocol.

### Probe intensities

The intensity values obtained from the scanned microarray images are well approximated using the function [1,58-60](2)

where *p *is the probe index. *I*^*max *^and *I*^*min *^denote the maximum and minimum intensity values referring to saturation of the probe spots and to the optical background, respectively. Both values are approximated as chip-specific constants not depending on individual probe properties. The linearized signal, *L*_*p*_, additively decomposes into contributions owing to non-specific and specific binding, , which linearly scale with the respective concentration of non-specific and specific transcripts,  with *h *= *N, S*.

According to Eq. 2, probe intensities on microarrays are hyperbolic functions of transcript abundance which saturate in the limit of large values *L*_*p *_→ ∞ and level off to a non-specific background intensity at vanishing *L*_*p *_→ 0. Specific and non-specific hybridization imply different probe-target duplexes which are stabilized by partly different base pair interactions [16]. The analysis of probe intensities in terms of sequence effects therefore raises two potential problems which have to be taken into account: Firstly, the sequence effect nonlinearly scales with the transcript concentration, and secondly, it decomposes into two contributions due to specific and non-specific hybridization on the level of base pairings.

### Positional dependent sensitivity model

The linearized signals can be split into sequence-independent and -dependent terms according to(3)

with *h *= *N, S *and log  = const + [*h*]. Here we omit the probe index for the sake of convenience.  denotes the 25meric sequence of the selected probe in terms of a string of 25 letters (Affymetrix microarrays use a common probe length of 25 nucleotides. '25' therefore denotes the probe length). The chip average of the sequence-dependent term is selected to be centered about zero, ⟨*δ A*^*h*^(ξ)⟩_*chip *_= 0. The sequence-independent term  is directly related to the target concentration of specific or nonspecific transcripts, [*h*] = [*N*], [*S*].

We further use the convention ξ^*k*, *k*+*r*-1 ^to assign the subsequence of *r *adjacent nucleotides starting at position *k *in *ξ*. The sequence effect is modelled using the sum of sensitivity terms over all sequence positions [18,32,47](4)

The sensitivity profiles  depend on base tuples (*b*_*r*_)*k *= (*B*_1 _⋯ *B*_*r*_)*k *(with the *B*_*i *_∈ *{A, T, G, C }*, 1 ≤ *i *≤ *r*) of length r with its first base at position k of the probe sequence. For example, (*GGG*)_1 _denotes a sequence motif containing three adjacent guanines beginning at sequence position 1. The parameter *r *specifies the rank of the model. Thus, *r *= 1 ... 4 refers to the single nucleotide (N), nearest neighbour (NN), next nearest neighbour (NNN) and quadruple (NNNN) models, respectively.

Integral sensitivities are calculated by summing up the positional dependent values either over all sequence positions or over a positional range that was selected, for example, to exclude the region of the (*GGG*)_1_-effect:(5)

### Analyzing absent probes

Equation 2 simplifies into *I*_*p *∈ *N *_- *I*^*min *^≈  for the special case of predominantly non-specific binding far below saturation,  ≪  ≪ *I*^*max*^. Restricting our basic analysis to this regime, we ensure linearity of the intensity response and homogeneous probe-target interactions. The latter are mainly governed by canonical Watson-Crick pairings [16].

We selected the subensemble of probes meeting these conditions using the recently developed hook method [31,32]. For each particular chip, it processes intensity combinations of paired PM and MM probes to estimate the detection limit of the specific signal  and classifies the probesets into 'absent' (*p *∈ *N*) or 'present' (*p *∉ *N*) ones. An analogous present/absent calling concept is used by the Affymetrix standard analysis [61]. Table 2 shows that more than 40% of all probesets on the studied arrays are called 'absent', providing a sufficient number of probe intensities to adequately fit the model (see below).

The hook method also estimates the maximum saturation intensity *I*^*max *^of the considered arrays [31,32]. Table 2 shows that their values exceed the mean intensity of the 'absent' probes by more than two orders of magnitude as required for linear approximation.

The raw intensities were corrected for the optical background using the Affymetrix zone algorithm which calculates the correction term as a function of the probe position using the 2% lowest probe intensities (see [61]).

### Analyzing specific probes

The ensemble of present (i.e. not-absent) probes refers to signals which partly or completely originate from specific hybridization. Binding characteristics are known to be different for specific binding [15]. We apply the hook method to filter out probe sets which hybridize predominantly with specific transcripts, (*p *∈ *S*), and to correct their intensities for the effect of saturation (see [32] for details). In the following we omit the superscript '*N *' or '*S*' with the understanding that the intensities are separately analyzed for specific and nonspecific hybridization.

### Estimating the sensitivity profiles

We define the experimental sensitivity of each probe as the deviation of the logged linearized signal from its average over all probes of the respective probeset [1](6)

After insertion of Eqs. (3) and (4) into (6) and making use of log(*L*_0_) = ⟨log(*L*_0_)⟩_*pset *_we get the theoretical sensitivity of each probe(7)

with the Kroenecker function *δ *(*x, y*) = 1 for x = y and *δ *(*x, y*) = 0 otherwise.  is the probability to find motif *b*_*r *_at sequence position k among the probes of the considered probeset. Note that the transcript concentration (specific and non-specific) is assumed to be constant for each probeset because each probe within the set targets the same transcipt. This condition cancels the term log(*L*_0_) in Eq. (3).

The sensitivity profiles are estimated using multiple linear regression. It minimizes the sum of squared residuals [32](8)

with *RES *= (*Y*^*exp *^- *Y*^*theo*^) by optimizing *σ*_*k*_(*b*_*r*_) for all 4^*r*^·(25 - *r *+ 1) base tuples (*b*_*r*_)_*k*_. The sum runs over all relevant probes *p *∈ *N *or *p *∈ *S *(#*p *defines the respective number of probes). The obtained sensitivity terms meet the center condition  for each sequence position *k*.

### Model-rank assessment

The number of independent parameters of the positional dependent sensitivity model increases with the rank according to(9)

providing #*σ *(*r*) = 76, 361, 1450 and 5611 for *r *= 1 ... 4, respectively.

The significance of increasing the rank ((*r *- 1) → *r*) of such nested models can be tested using the F-statistics(10)

It follows the F-distribution with the degrees of freedom *df*(*r*) = #*p *- #*σ*(*r*) + 1 and allows to estimate the significance of model extension in terms of a p-value. Usually one gets *df *≊ #*p *because the number of probes (> 10^5^) largely exceeds the number of model parameters (≲10^3^). One consequence of the large number of probe values is that essentially each improvement of the fit with *F *> 1.5 is judged as significant with *p *< 10^-2 ^for *df *> 10^5^.

Eq. (10) applies under the assumption of normally distributed, independent residuals. We found that systematic errors partly contribute to the estimated SSR questioning the applicability of the F-test. We therefore use the F-values as a simple empirical measure characterizing the improvement of the fits.

### Sequence motif assessment

We assume that a model of rank r applies with different quality to different sequence motifs of length *s *at position *k*, (*b*_*s*_)_*k*_. Note that the length of the motif *s *is independent of the rank of the model. For example, triple motifs (*s *= 3; e.g., GGC) can be analyzed either using the nearest neighbor model (*r *= 2; i.e., GG+GC) or the next-nearest neighbor model (*r *= 3; i.e., GGC). To assess the fit quality in a motif specific fashion we collect all probe sequences which contain (*b*_*s*_)_*k *_into class *p*((*b*_*s*_)_*k*_) with #*p*((*b*_*s*_)_*k*_) members per chip and define the motif-specific SSR in analogy with Eq. (8)(11)

One can subsume all motif effects independently of their position by substituting (*b*_*s*_)_*k *_→ *b*_*s *_in Eq. (11) to get the total SSR of tuple *b*_*s*_, *SSR*(*b*_*s*_).

Note that the total SSR (Eq. 8) is given as the weighted sum of the motif-specific SSR(12)

where *f*(*b*_*s*_)_*k *_= #*p*((*b*_*s*_)_*k*_)*/*#*p *denotes the fraction of probes containing the respective motif.

Motif-specific F-values *F *(*r, b*_*s*_) and *F *(*r*, (*b*_*s*_)_*k*_) can be calculated for the respective SSR and with the respective substutions for the number of probes (#*p *→ #*p*(*b*_*s*_); #*p*((*b*_*s*_)_*k*_)) to judge the improvement of the model with respect to the chosen sequence motif. The number of relevant parameters is given by the number of model tuples *b*_*r *_required to describe the sequence motif *b*_*s *_at all positions for the positional independent case. It provides #*σ *(*b*_*s*_) = (*s *- *r *+ 1)·(25 - *s *+ 1) and #*σ *((*b*_*s*_)_*k*_) = *s *- *r *+ 1 for the positional dependent and indepentent cases, respectively.

### Quality of fit and standard error

The positional and motif-specific SSR (Eq. (11)) estimate the contribution of a subensemble of probes containing the motif (*b*_*s*_)_*k *_to the total sum of squared errors after fitting the positional dependent sensitivity model of rank r to the whole ensemble of considered probes (Eq. (12)). Ideally, the residuals scatter with equal variance and center zero for each chosen motif. To detect and to estimate systematic biases of the fits in a motif specific fashion we calculate the squared sum of the respective residuals to judge the quality of the fits for each considered sequence motif,(13)

Ideally one expects *QF *(*r*, (*b*_*s*_)_*k*_) = 0 for centered distributions of the residuals. Non-zero values *QF *(*r*, (*b*_*s*_)_*k*_) ≠ 0 thus indicate systematic deviations of the fits of the model of rank *r *with respect to motif (*b*_*s*_)_*k*_.

The motif-specific variance of the residuals and the respective standard error are given by

and(14)

The standard error allows to estimate the confidence level of the positional dependent sensitivity terms *σ*_*k*_(*b*_*s*_).

### The NN+GGG hybrid rank model

The hybrid rank model basically uses the positional dependent NN-model to correct the probe intensities according to Eq. (4). In addition it applies positional dependent NNN-terms for intensity effects which are inadequately corrected by the NN-model. In our special application the model considers GGG-terms to correct the intensities for effects due to degenerated guanines.

The algorithm works as follows:

1) The set of predominantly non-specifically hybridized probe sets, the so-called 'absent' or N-subset, is identified using the hook method [31,32] (see above).

2) The N-subset is further split into two sub-ensembles not-containing and containing triple-G motifs, *PS*_*NN *_and *PS*_*GGG*_, respectively. They are subsequently corrected in two steps for sequence effects:

2a) The *P S*_*NN *_sub-ensemble is used to train the NN model by multiple linear regression of the data using Eq. (6) - (8) with *r *= 2. The fit provides the basal set of NN-terms *σ*^*NN *^= *σ*_*k*_(*b*_2_).

2b) Each probe set of the second *P S*_*GGG *_sub-ensemble contains at least one probe with at minimum one motif of three consecutive guanines. Eq. (3) rewrites for these probes into(15)

where *δ A*^*NN *^(*ξ*) is given by Eq. (4) with *r *= 2 and the set of NN-terms estimated in step 2a. The excess correction term *δ A*^*GGG*^(*ξ*) considers the effect of the critical motif in the probe sequences in analogy with Eq. (5)(16)

With Eq. (6) one gets the theoretical sensitivity(17)

 denotes the basal sensitivity which is calculated using Eq. (7) and the basal set of NN-terms estimated in step 2a. After minimizing Eq. (8) one gets the profile of excess terms *σ*_*k*_(*GGG*).

3) The corrected linearized intensities of the probes of the *P S*_*NN *_- and *P S*_*GGG*_-subsets, *L*_0_, are calculated after rearrangement of Eqs. (3) and (15), respectively.

4) The present probes not included in the N-sub ensemble are corrected as described previously [31,32]. In short: A NN-model of rank *r *= 2 is parametrized using the probe sets which are hybridized to more than 80% with specific transcripts. They are then corrected using this model. Probe sets with a fraction of specific-hybridization of less than 80% are corrected by a weighted combination of the sensitivity profiles referring to specific and non-specific hybridization determined in step 2.

5) The sensitivity-corrected intensity data are exported in the standard *.cel file format. The corrected signal values can then be feed into standard GeneChip preprocessing programs for further improvement and/or downstream analysis.

The program can be downloaded from our website http://www.izbi.uni-leipzig.de/downloads_links/programs/hook.php.

## Competing interests

The authors declare that they have no competing interests.

## Authors' contributions

HB and MF conceived and designed the study, analyzed the data and wrote the manuscript. All authors have read and approved the final manuscript.

## Supplementary Material

Additional file 1 The additional text provides a list of the datasets studied, the frequencies of triple motifs on selected array types and the positional sensitivity profiles of specific and nonspecific hybridization for three selected hybridizations. Click here for file
