# Supplementary material for: G-stack modulated probe intensities on expression arrays - sequence corrections and signal calibration
Source: BMC Bioinformatics. 2010 Apr 27;11:207. doi: 10.1186/1471-2105-11-207 (PMC2884167; doi:10.1186/1471-2105-11-207)
Supplement: Additional file 1 — The additional text provides a list of the datasets studied, the frequencies of triple motifs on selected array types and the positional sensitivity profiles of specific and nonspecific hybridization for three selected hybridizations. [file 1471-2105-11-207-S1.PDF]

Supplemental Material: G-stack modulated probe intensities on expression arrays - sequence corrections and signal calibration

[1]Interdisciplinary Centre for Bioinformatics, University Leipzig, Germany
[2]Leipzig Interdisciplinary Research Cluster of Genetic Factors, Clinical Phenotypes and Environment (LIFE), University Leipzig, Germany
[3]Max-Planck-Insitute for Mathematics in Sciences, Leipzig, Germany
[4]Fraunhofer Institut for Cell Therapy and Immunology, Leipzig, Germany
[5]Institute for Theoretical Chemistry, University of Vienna, Vienna, Austria
[6]The Santa Fe Institute, Santa Fe, New Mexico, USA

Email:

*Corresponding author

Supplementary Table 1: Microarray data studied. GSExxxx and GSMxxxx are the accession numbers of datasets downloaded from the GEO repository http://www.ncbi.nlm.nih.gov/geo/.

Chip-type	Employed publicly available datasets
ENCODE	GSE2800
Yeast_2	GSE9302
ChipChIP	GSE2987
MG430_2	GSE12545
Zebrafish	GSE5048
EColi_2	GSE4724
CElegans	GSE6547
Rice	GSE6893
Chicken	GSE12268
ATH1-121501	GSE7432
Rat230_2	ArrayExpress E-TABM-536
MG430A	GSM154799, GSM355022, GSM366810
DrosGenome1	Fruitfly time series (http://camda.bioinfo.cipf.es/camda08/contest_dataset)
MG74A	GSM104601, GSM34328, GSM4310
HGU133A	Affymetrix Latin Square HG-U133A (http://www.affymetrix.com/support/technical/sample_data/datasets.affx)
HGU95A	Affymetrix Latin Square HG-U95A (http://www.affymetrix.com/support/technical/sample_data/datasets.affx)
30 Mouse MG430_2 Chips	GSM172403, GSM176889, GSM177368, GSM178084, GSM187846, GSM211338, GSM211425, GSM237785, GSM238367, GSM250880, GSM252214, GSM264815, GSM280709, GSM282803, GSM311514, GSM313208, GSM315604, GSM318915, GSM325421, GSM326978, GSM326998, GSM337788, GSM337834, GSM432906, GSM443776, GSM455430, GSM53318, GSM94768

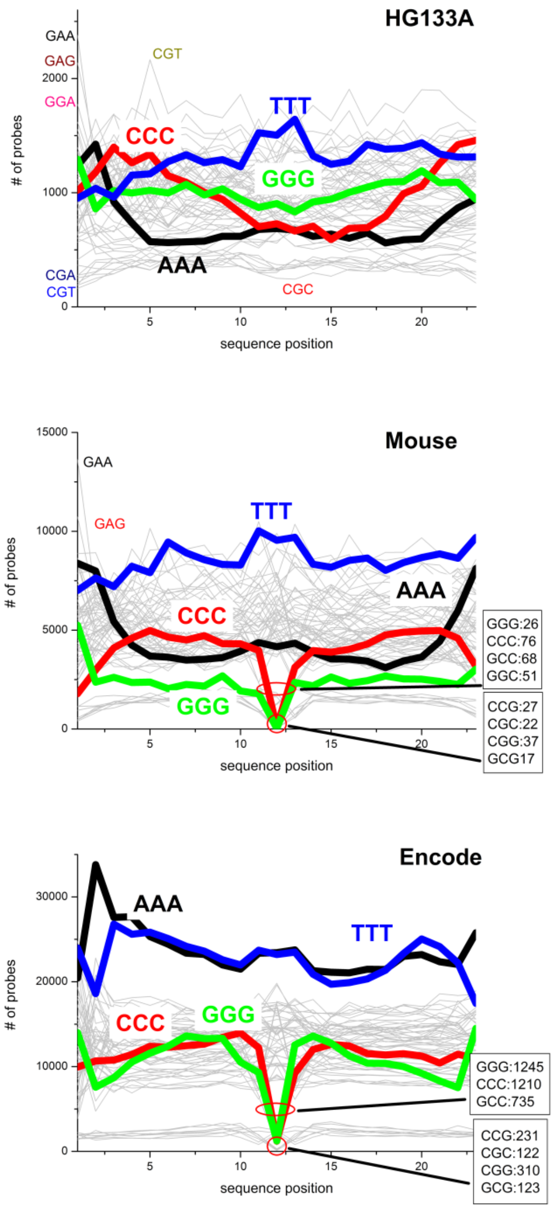

Supplementary Figure 1: Frequency of triple motifs. The figures show the positional dependence of the triple motifs in the probe sequences of three selected array-types. Homo-motifs are highlighted by thick colored curves. Note the partly different scaling of the ordinates. Rare triple motifs at position $k = 13$ are explicitly given in the boxes together with the respective number of probes containing the motif. For example, only 27 probes on the MG 430 2.0 array contain CCG-triples starting at $k = 13$. Note that these rare motifs give rise to large spikes of the respective standard errors for the triple terms.

HG133A_S

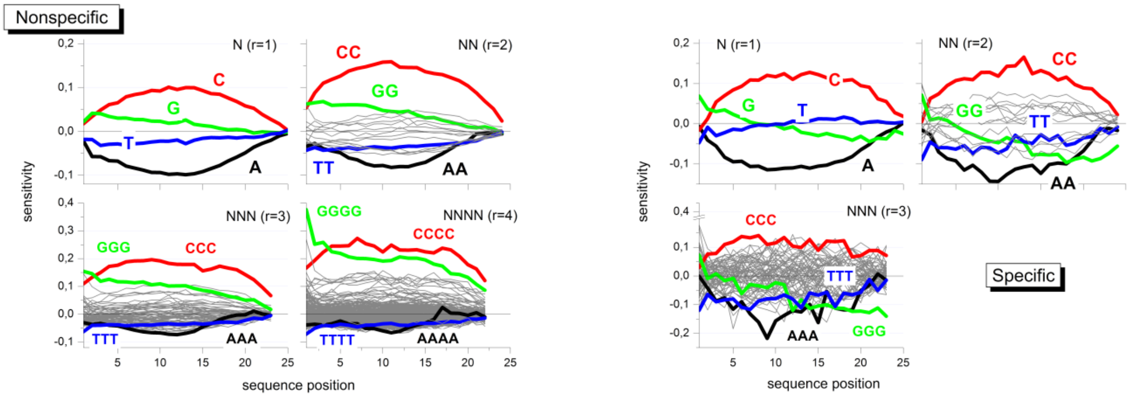

Mouse

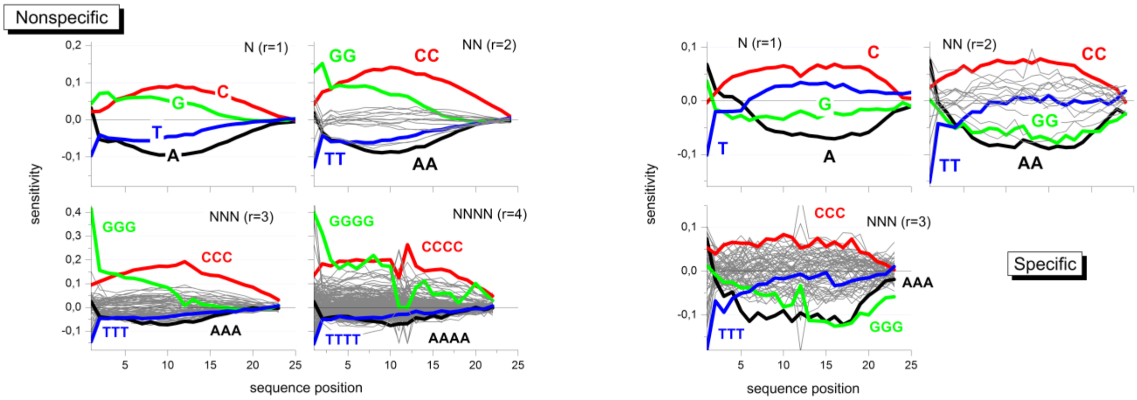

ENCODE

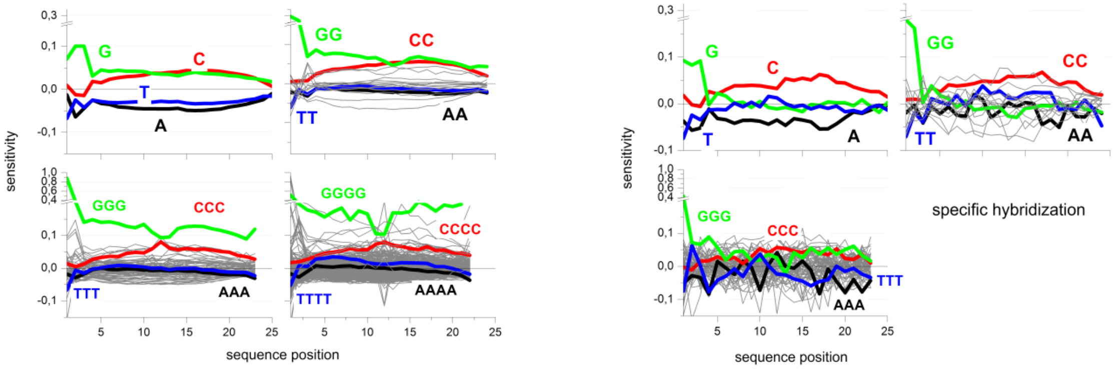

Supplementary Figure 2: Positional dependent sensitivity motifs of specific and nonspecific hybridization. The figures show the sensitivity profiles of non-specific (left) and specific (right) hybridization of different rank for three of the studied arrays. Homo-motifs are highlighted by thick colored curves. Note the partly different scalings of the ordinates. The data sets are assigned in Table 2 of the paper.
